# Single-Cell RNA Sequencing Characterizes the Molecular Heterogeneity of the Larval Zebrafish Optic Tectum

**DOI:** 10.3389/fnmol.2022.818007

**Published:** 2022-02-10

**Authors:** Annalie Martin, Anne Babbitt, Allison G. Pickens, Brett E. Pickett, Jonathon T. Hill, Arminda Suli

**Affiliations:** ^1^Department of Cell Biology and Physiology, Brigham Young University, Provo, UT, United States; ^2^Department of Microbiology and Molecular Biology, Brigham Young University, Provo, UT, United States

**Keywords:** optic tectum, zebrafish, molecular characterization, single-cell RNA sequencing, cell type identification

## Abstract

The optic tectum (OT) is a multilaminated midbrain structure that acts as the primary retinorecipient in the zebrafish brain. Homologous to the mammalian superior colliculus, the OT is responsible for the reception and integration of stimuli, followed by elicitation of salient behavioral responses. While the OT has been the focus of functional experiments for decades, less is known concerning specific cell types, microcircuitry, and their individual functions within the OT. Recent efforts have contributed substantially to the knowledge of tectal cell types; however, a comprehensive cell catalog is incomplete. Here we contribute to this growing effort by applying single-cell RNA Sequencing (scRNA-seq) to characterize the transcriptomic profiles of tectal cells labeled by the transgenic enhancer trap line *y304Et*(cfos:*Gal4*;UAS:*Kaede*). We sequenced 13,320 cells, a 4X cellular coverage, and identified 25 putative OT cell populations. Within those cells, we identified several mature and developing neuronal populations, as well as non-neuronal cell types including oligodendrocytes and microglia. Although most mature neurons demonstrate GABAergic activity, several glutamatergic populations are present, as well as one glycinergic population. We also conducted Gene Ontology analysis to identify enriched biological processes, and computed RNA velocity to infer current and future transcriptional cell states. Finally, we conducted *in situ* hybridization to validate our bioinformatic analyses and spatially map select clusters. In conclusion, the larval zebrafish OT is a complex structure containing at least 25 transcriptionally distinct cell populations. To our knowledge, this is the first time scRNA-seq has been applied to explore the OT alone and in depth.

## Introduction

The superior colliculus (SC) is a highly laminated multisensory processing hub located in the mammalian midbrain that receives sensory input of various modalities and is involved in visual, motor, and sensory pathways (Krauzlis et al., 2013; Stein and Stanford, 2013), as well as perceptual decision-making (Jun et al., 2021). Despite studies dating back to the 1970s (Basso and May, 2017), knowledge concerning the different neuronal cell types comprising SC microcircuitries and their respective functions is limited. The homologous optic tectum (OT), found in non-mammalian vertebrate species such as zebrafish, provides an excellent opportunity to study the SC in a more physically and genetically accessible model organism (Del Bene and Wyart, 2012). Similar to the mammalian SC, the OT has the critical role of receiving sensory input. It is responsible for visually guided behaviors such as phototaxis, prey capture, obstacle avoidance, and predator escape (Wong, 1999; Gahtan et al., 2005; Nevin et al., 2008, 2010; Fero et al., 2011; Lowe et al., 2013; Vanwalleghem et al., 2017). In larval zebrafish, the tectum can be broadly divided into two regions: the periventricular layer (PVL), where the majority of tectal cell bodies lie; and the neuropil (NP), which consists primarily of neurites. Within the neuropil, several layers can be further identified: the stratum fibrosum marginale (SM), stratum opticum (SO), stratum fibrosum et griseum superficiale (SFGS), stratum griseum centrale (SGC), and the stratum album centrale (SAC) (Nevin et al., 2010). The most superficial layer, the SM, does not receive visual input, while all deeper layers are heavily innervated by retinal ganglion cells (RGCs) which project from the contralateral eye and terminate in specific tectal laminae (Xiao et al., 2011; Robles et al., 2013, 2014; Heap et al., 2018). Instead, the SM receives input from the torus longitudinalis (Meek and Schellart, 1978; Perry et al., 2010) while the PVL receives afferent input from the Raphe nucleus and cerebellum (Yokogawa et al., 2012; Filosa et al., 2016). A hypothalamic influence on the OT has also been uncovered, as neurons within the rostral hypothalamus project to the SFGS and an area between the SGC and SAC (Heap et al., 2018).

Although teleost fish lack a visual cortex, the tectum’s role in complex visually evoked behaviors, combined with recent evidence of an ability to respond to both auditory and water flow stimuli (Gahtan et al., 2005; Thompson et al., 2016; Vanwalleghem et al., 2017), attests to the functional similarities between the OT and the SC, qualifying it as a valuable, albeit simpler, model for understanding SC microcircuitry. A complete understanding of the OT at a functional and cellular level requires a comprehensive catalog of tectal neurons including their morphologies, synaptic partners and modulation, along with their molecular signatures. Such an undertaking in teleosts begun as early as the 1970s, as Meek and Schellart identified the morphologies and spatial localizations of 14 neuron types in the adult goldfish optic tectum via Golgi-stain labeling (Meek and Schellart, 1978). In the zebrafish optic tectum, significant strides have been made to characterize cell type diversity using a variety of molecular and genetic methods. Recently, a *Gal4* enhancer trap screen generated over 150 stable transgenic lines, 13 of which showed specific tectal expression (Scott et al., 2007). Three of those 13 lines were further surveyed, resulting in the identification of multiple neuronal morphologies closely resembling those previously found in the adult goldfish optic tectum (Scott and Baier, 2009). Further tectal diversity studies have resulted in the characterization of previously unidentified cell types within the optic tectum, as well as cell types that corroborate previous studies in other teleosts (Del Bene et al., 2010; Robles et al., 2011; Barker et al., 2020; DeMarco et al., 2020). These cell type diversity studies (which combine functional imaging, electrophysiological recordings, and neurotransmitter typing) together with the recent publication of a larval zebrafish brain atlas (Kunst et al., 2019), constitute a wealth of knowledge surrounding the zebrafish optic tectum. However, a key piece of the comprehensive catalog is missing: the molecular transcriptomes of tectal cells. In 2020, a landmark study began this work by generating a transcriptomic single-cell atlas of zebrafish development, highlighting gene expression changes between 1 and 5 days-post-fertilization (dpf) (Farnsworth et al., 2020). Undoubtedly an invaluable developmental resource, the comprehensive nature of this study precludes an in-depth analysis of the optic tectum, as it (1) looks at whole-animal gene expression changes and (2) is limited to the first few days of development. The visual system develops rapidly in zebrafish, as RGCs reach the tectal neuropil as early as 48 hpf, and by 3 dpf optokinetic responses are in place; however, the optic tectum undergoes rapid development between 3 and 7 dpf as neurite growth and synaptogenesis continue until approximately 7–8 dpf when the tectum is considered functionally mature (Niell et al., 2004; Niell and Smith, 2005; Pietri et al., 2017). Particularly as most functional and morphological studies are conducted between 5 and 10 dpf, in depth information on the molecular transcriptomes in the OT during this time is essential.

In this study, we contribute to optic tectum characterization by fulfilling the critical need for tectal cell type classification according to gene expression profiles obtained via single-cell RNA sequencing. Single-cell RNA sequencing (scRNA-seq) technology is a relatively novel method for transcriptional analysis that has been gaining favor over the last several years. A key benefit of this technology is the ability to quantify meaningful cell-to-cell gene expression variability in diverse tissues, allowing for the creation of comprehensive cell catalogs (Chen et al., 2019; Luecken and Theis, 2019). scRNA-seq provides direct access to cellular transcriptomes, and in recent years has been successfully utilized to characterize the zebrafish lateral line, retinal ganglion cells, and habenula (Pandey et al., 2018; Lush et al., 2019; Kölsch et al., 2021). Using the transgenic *y304Et*(cfos:*Gal4*;UAS:*Kaede*), an enhancer trap line which comprehensively labels the optic tectum (Marquart et al., 2015), we utilized scRNA-seq technology to identify and characterize 25 putative cell populations within the larval zebrafish optic tectum. These transcriptomic data provide an important groundwork for the complete comprehensive characterization of the OT, as we describe the neurotransmitter identity, developmental state, potential synaptic partners, and gene expression profiles of these molecularly distinct cell populations. To our knowledge, this is the first time scRNA-seq technology has been applied to the zebrafish optic tectum alone and in depth.

## Materials and Methods

### Resource Availability

Further information and requests concerning resources and reagents should be directed to the lead corresponding author, Arminda Suli (asuli@byu.edu).

#### Materials

HCR RNA-FISH probe set sequences are the intellectual property of Molecular Instruments (Los Angeles, CA). We are glad to direct sequence inquiries to their team.

#### Raw Sequence Data

Raw data generated from this study will be available at NCBI’s Sequence Read Archive at time of publication (SRA Accession Number: PRJNA779441).

### Zebrafish Lines and Husbandry

All experiments were performed according to guidelines established by the IACUC review board at Brigham Young University (IACUC Protocol Number: 19-0901). Larvae from the *y304Et*(cfos:*Gal4*;UAS:*Kaede*) enhancer trap line (Marquart et al., 2015) were raised on a 14 h:10 h light:dark cycle and maintained at 28.5°C until 7 dpf, at which point they were either harvested for scRNA-seq or fixed in 4% paraformaldehyde preparatory to fluorescent *in situ* hybridization.

### Sample Preparation

15,922 larval zebrafish cells were sequenced from two temporal replicates. Sample preparation across replicates was consistent regarding personnel, equipment, protocol, and sequencing technology.

For each replicate, 7 dpf larvae were anesthetized using tricaine (1:10). Heads were collected via a single cut at the posterior hindbrain and kept on ice for an average of 30 min. They were dissociated at 28.5°C in 1 mL 1% Trypsin-EDTA, with gentle trituration every 5 min for 1 h. Dissociated cells were filtered with a 70 μm Cup Filcon Filter (BD Biosciences, San Jose, CA, United States) and washed with ice cold DPBS (centrifugation at 5,000 rpm for 5 min at 4°C). Cells were stained with DRAQ5 (1:1,000) (biostatus, United Kingdom) and DAPI (1:1,000) to gate dead cell populations. Fluorescent activated cell sorting (FACS) for live Kaede^+^ cells (Supplementary Figure 1) was performed at Brigham Young University (Provo, UT, United States) using a FACS Aria Fusion Cell Sorter (BD Biosciences, San Jose, CA, United States). Cells were sorted at a reduced flow rate immediately into methanol to preserve gene expression and pooled together for rehydration and resuspension in preparation for sequencing. Methanol fixed cells were rehydrated via centrifugation in a swinging bucket rotor at 2,000 g for 5 min at 4°C and resuspended at a concentration of 1,000 cells/μL in DPBS with 0.09% BSA.

### Library Preparation and Sequencing

Library preparation and sequencing were performed externally at the University of Utah Huntsman Cancer Institute (Salt Lake City, UT, United States) on the 10X Chromium single cell platform (10X Genomics, Pleasanton, CA, United States) using the scRNA-seq 3′ v3.1 Next GEM library preparation pipeline. For each temporal replicate, approximately 30,000 FAC-sorted, methanol-fixed, Kaede^+^ cells were used as input for sequencing; with maximum targeting of 10,000 cells and a sequencing depth of 200 M reads.

### Read Alignment and Quantification

Raw reads were aligned to version 10 of the zebrafish genome using the 10X Genomics Cell Ranger 4.0.0^1^ pipeline. Replicate 1 generated 6,629 barcodes and replicate 2 generated 9,293; each set of barcodes was used to generate a count matrix for downstream analysis (SRA Accession Number: PRJNA779441).

### Batch Correction and Quality Control

Quality control was performed following the workflow outlined by the Harvard Chan Bioinformatics Core in their Single-cell RNA-seq data analysis workshop (Harvard Chan Bioinformatics Core, 2020). Initial quality control was performed to assess the likelihood of doublets, where two cells are encapsulated and sequenced together, by examining the distribution of genes detected per cell. Datasets with significantly higher than average numbers indicate unusual complexity and often results in a bimodal distribution, suggesting the presence of doublets. An initial qualitative check was performed, and a normal distribution was observed with an average of 729 genes detected per cell. As a result, filtration of cells with a high number of reads was not deemed necessary, as recommended by the Harvard Chan Bioinformatics Core. To further mitigate the effects of low-quality cells on downstream analyses, cells fulfilling one or more of the following criteria were removed: (1) fewer than 500 UMIs, (2) fewer than 300 genes, and (3) greater than 10% mitochondrial gene content. These parameters identified and removed 1,218 low-quality cells, yielding a final dataset of 14,704 cells. Cell cycle scoring was completed as recommended, and regression of associated genes was not deemed necessary (Supplementary Figure 2; Harvard Chan Bioinformatics Core, 2020).

Datasets were considered replicates, and therefore not hypothesized to display confounding technical variation. Datasets were examined for batch effects by merging into a single matrix with 15,922 genes, applying a standard analysis workflow, and visualizing the resulting PCA and UMAP plots. A significant amount of overlap between datasets was observed on both PCA and UMAP plots (Supplementary Figures 3A,B), suggesting that merging datasets did not introduce meaningful variation. To further confirm a lack of batch effects, we determined the expression profile of the canonical habenula marker, *gng8*, and show that habenula cells cluster together across datasets rather than by dataset (Supplementary Figure 3C). For these reasons, batch correction was not deemed necessary, as technical variation between datasets was not sufficient to obscure true biological variation.

Following quality control, all downstream analyses were implemented using the R package Seurat (v4.0.3) (Hao et al., 2021), following the workflows described on the Satija lab website^2^. Using SCTransform (Hafemeister and Satija, 2019), data normalization was accomplished by calculating Pearson residuals for the remaining UMI counts via regularized negative binomial regression. SCTransform, a framework for the normalization and variance stabilization of UMI data, has been shown to outperform previous methods by mitigating the effects of technical characteristics such as sequencing depth while preserving biological heterogeneity (Hafemeister and Satija, 2019). The resulting data matrix was then centered in preparation for dimensional reduction.

### Dimensional Reduction

Pearson residuals were used as direct input for PCA. Forty-three principal components (PCs) were used for dimensional reduction, determined by calculating the percent of variation associated with each PC, and selecting the PC where: (1) 90% of variation was cumulatively explained, and (2) the variation associated with that PC is less than 5%; as recommended by the Harvard Bioinformatics Core (Harvard Chan Bioinformatics Core, 2020). For visualization purposes, an additional round of dimensional reduction was performed using Uniform Manifold Approximation and Projection (UMAP) post cluster identification.

### Cluster Identification and Annotation

Unsupervised clustering was performed with the Seurat function FindClusters(), using the Louvain algorithm with multi-level refinement. Candidate gene markers for each cluster were identified via Seurat’s FindAllMarkers() which implements the MAST test for differential expression. MAST employs a hurdle model tailored to scRNA-seq data by addressing specific characteristics such as stochastic dropout and bimodal expression (Finak et al., 2015). Genes were considered unique markers if they were both highly (>1 log2fc) and widely (>50% of cells) expressed within the cluster of interest compared to all other cells.

Clusters representing the habenula, epiphysis, heart, and olfactory system were identified via known marker genes compiled using the ZFIN database^3^ (Supplementary Figures 4A,B, 5A,B and Supplementary Table 1). All cells in non-tectal clusters were excluded from downstream analyses.

Similarity of OT clusters was calculated using the Seurat function BuildClusterTree(), which constructs a phylogram relating average cells from each cluster based upon a distance matrix constructed in PCA space (Hao et al., 2021).

### Confirmation of *Gal4* Insertion and Kaede Detection

The *Gal4* insertion site was obtained via personal communication with Harold Burgess, who provided us with the *y304Et*(cfos:*Gal4*;UAS:*kaede*) enhancer trap line, and confirmed via PCR amplification (Supplementary Figure 6A). *kaede* detection was confirmed in all clusters (Supplementary Figure 6B), along with expression of the three most proximal genes to the *Gal4* insertion site namely: *ctnnb1*, *jupa*, and *cmtm8b* (Supplementary Figures 6C–F). *ctnnb1*, was previously found to be expressed in areas including OT and habenula (Osborn et al., 2014). *jup* (*jupa*) is expressed in the olfactory organ (Thisse et al., 2001; Thisse and Thisse, 2004), while *cmtm8b* is predicted to be involved in myelination (ZFIN^3^ database).

### Gene Ontology Analysis

Gene ontology analysis identifies upregulated biological processes through an input list of genes, and retrieves the GO terms describing the biological processes(s) each gene is involved in Ashburner et al., 2000Ashburner et al., 2000). Whole data-set gene ontology (GO) analysis was conducted on all significant (*p* < 0.05) differentially expressed genes (DEGs) as identified by the MAST test (Supplementary Tables 2, 3). Note that although the same statistical method was used to determine differentially expressed genes as to nominate candidate cluster markers, the parameters for marker genes were significantly more stringent. For the purposes of GO analysis, a DEG for any given cluster is present in at least 25% of cluster cells and expressed at least 0.25 log2fc higher in that cluster as opposed to all other cells. GO analysis was not conducted on populations annotated as non-tectal, and any duplicate genes were removed from the final list of DEGs (2,222 genes) which were then used as input. Using PANTHER GO^4^ (Ashburner et al., 2000), an online tool maintained by The Gene Ontology Consortium (2021), we conducted a PANTHER Overrepresentation Analysis for biological process complete annotations using Fisher’s Exact Test with FDR correction (Mi et al., 2019). This identifies significant biological processes within the input gene list and provides an effective preliminary tool to explore gene expression data.

### Gene Expression Profile Analysis

To annotate clusters containing mature neurons, it was reasoned that only functioning, post-mitotic neurons would be actively involved in neurotransmission. Genes involved in various aspects of neurotransmission, such *vamp1* and *sv2a* (vesicle docking/fusion) were manually curated according to their functional designation on ZFIN^3^. For a complete list of neurotransmission processes and their respective genes refer to Supplementary Table 4. Mature annotation was further confirmed via characterization of neuronal profiles.

To characterize mature and developing neuronal profiles, we performed DEA of genes unique to GABAergic, glutamatergic, dopaminergic, glycinergic, cholinergic, serotonergic, and histaminergic neurons (Supplementary Table 7). Unique genes for each neurotransmitter were manually curated via ZFIN^3^ and characterized as either presynaptic (describing the neuron itself) or postsynaptic (describing the neuron’s synaptic partners). Presynaptic markers were defined as genes responsible for aspects of neurotransmission such as synthesis, packaging, and presynaptic reuptake, so long as they are unique to a neurotransmitter. To illustrate this process, we demonstrate the characterization of glutamatergic transmission. Unique presynaptic markers include genes such as *slc17a7a/b* (VGLUT1), *slc17a6a*/*b* (VGLUT2), and *slc17a8* (VGLUT3). These genes, present only in glutamatergic neurons, encode vesicular glutamate transporters responsible for loading glutamate into vesicles prior to release at the synaptic cleft. In contrast, *slc1a2a/b* (GLT1) encodes an excitatory amino acid transporter (EAAT) responsible for clearing excess glutamate from the synaptic cleft. However, it cannot be used as a unique presynaptic marker for glutamatergic neurons as glial cells also aid in glutamate removal via GLT1 and other EAATs. Postsynaptic markers for glutamatergic transmission include genes such as *grin1a* and *gria1a*, which build NMDA and AMPA receptors, (respectively), present only on the postsynaptic cell membrane.

Oligodendrocyte, microglia and radial glial populations were annotated based upon previously known markers such as *mbpa*/*b*, *olig1*/*2*, and *mpeg1.1* (for a full list of markers see Supplementary Table 5). Transcription factors were obtained from the UniProt Consortium by curating all transcription factors for zebrafish (The UniProt Consortium, 2021). UniProt separates transcription factor records into categories based on review status; where reviewed factors are evaluated and manually annotated by UniProt curators based upon available literature (The UniProt Consortium, 2021). Only reviewed factors, 310 at time of analysis, were used in OT characterization; of these, only 257 factors were present within our data. To characterize transcription factor expression in OT populations, we examined the top differentially expressed, according to log2fc, and top overall expressed, according to percentage of expressing cells, within each population. The top two (differential expression) and top five (overall expression) are displayed for each population; duplicates are allowed but are only displayed once. All reviewed transcription factors are available in Supplementary Table 8.

Neurogenesis genes were obtained using AmiGO, a web-based software tool (Carbon et al., 2009) for browsing the Gene Ontology database (Ashburner et al., 2000; The Gene Ontology Consortium, 2021) and are available in Supplementary Table 6. ASD implicated genes were obtained from SFARI Gene, an evolving online database run by the Simons Foundation that evaluates clinical and biological data to score candidate risk genes and CNVs according to their association with ASD (Banerjee-Basu and Packer, 2010). These scores range from 1 to 3 and indicate a gene association of high confidence, strong candidate, and suggestive evidence, (respectively) (Banerjee-Basu and Packer, 2010). For the purpose of differential expression analysis, we curated zebrafish orthologs or paralogs for all SFARI genes with a score of 1 or 2, excluding those associated with syndromic ASD. This list comprised 316 genes, 298 of which had at least one known zebrafish ortholog or paralog, resulting in a total of 407 orthologs and paralogs that were used as input for differential expression analysis. All ASD implicated genes with their SFARI scores and orthologs are available in Supplementary Table 8.

### RNA Velocity Analysis

RNA velocity estimates future transcriptional cell states by analyzing the proportion of spliced and unspliced mRNAs of any given gene (La Manno et al., 2018). Position sorted BAM files, which contain binary versions of aligned sequence data, were generated by 10X Genomics Cell Ranger 4.0.0 for each replicate. These BAM files were used as input for velocyto.py^5^, which generates a loom file containing spliced and unspliced expression matrices. Each loom file was read using the Seurat function ReadVelocity(), converted into a Seurat object, and merged using the merge() function; the resulting object containing three expression matrices: spliced, unspliced, and ambiguous for all 15,922 cells. The object was then filtered to exclude cells previously removed during quality control of the original merged object.

Following filtration, RNA velocity was computed according to the Satija Lab tutorial for computing RNA velocity on Seurat objects (Satija Lab, 2019) which implements a wrapper around veloctyo.R. Velocity estimations were calculated using the Seurat RunVelocity() function and visualized using the original cluster designations and UMAP embedding obtained during the cluster identification.

### Hybridization Chain Reaction RNA Fluorescence *in situ* Hybridization

Kaede^–^ larvae from a *y304Et*(cfos:*Gal4*;UAS:*Kaede*;*tyr*^−/–^) in-cross were grown under standard conditions until 7 dpf and fixed in 4% paraformaldehyde (PFA) overnight at 4°C. Following fixation, larvae were washed in PBST (PBS + 10% Tween) for six 15-min increments and dehydrated for storage in methanol (MeOH) series (5 min each: 25% MeOH: 75% PBST; 50% MeOH: 50% PBST; 75% MeOH: 25% PBST; and 100% MeOH). Post dehydration, larvae were transferred into clean 100% MeOH and stored in –20°C. For use, larvae were rehydrated in reverse MeOH series (5 min. each: 100% MeOH; 5% MeOH: 25% PBST; 50% MeOH: 50% PBST; 25% MeOH: 75% PBST, 100% PBST), followed by 3 × 5 min washes in PBST and permeabilization at room temperature in 30 μg/mL Proteinase K for 45 min. Following rehydration and permeabilization, larvae were post-fixed with 4% PFA for 20 min followed by 5 × 5 min washes in PBST to remove the fixative.

HCR split-initiator probe sets were custom designed by Molecular Instruments^6^ using proprietary HCR methodology to detect and fluorescently label target RNA transcripts, while suppressing background automatically (Choi et al., 2018). To maximize targeting of *gng8*, alpha-*nrxn3a*, and *robo4*, probes were designed against shared regions of known variants found on NCBI Gene^7^ and the Ensemble database^8^ (Howe et al., 2021). Each probe set consisted of 20 split-initiator probe pairs per target, and all utilized a B1 amplifier, with a 546 nm fluorophore label. HCR RNA-FISH experiments were not multiplexed. Probe detection and amplification were performed as recommended by MI protocol for whole mount zebrafish larvae^9^ with the following optimizations: extended pre-hybridization time (3 h) and increased probe concentration (16 nM). Probe set sequences are considered intellectual property and are proprietary to Molecular Instruments; we direct all sequence inquiries to their technical team.

Following HCR RNA-FISH, larvae were stained with DAPI (1:500) for 3 h at room temperature, followed by 3 × 10 min washes in PBST. Larvae were then stored in 30% glycerol in PBST away from light at 4°C until imaging. Larvae were embedded dorsal side down in 1.25% low melting agarose on glass bottom culture dishes (MatTek, P35G-0-10C). Images were acquired on an inverted FV1000 Olympus confocal microscope (Olympus Corporation, Japan, Tokyo) with either a 20X or 40XW objective (Olympus, UAPON40XW340) using the 546 nm (B1 amplifier) and 405 nm (DAPI) lasers. Confocal image data was uploaded into Fiji (Schindelin et al., 2012) (ImageJ, Bethesda, MA, United States), where images were assigned reference colors and merged for visualization.

## Results

### Transcriptomic Profiling of 7 Days-Post-Fertilization Larval Zebrafish Shows the Optic Tectum Consists of at Least 25 Distinct Molecular Populations

**FIGURE 1:**
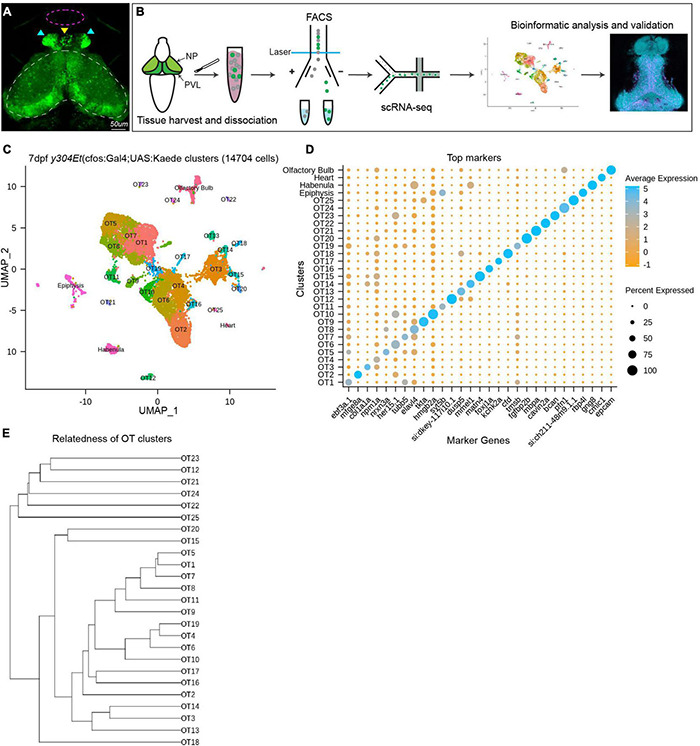
Analysis of scRNA-seq data reveals 25 distinct populations within the larval zebrafish optic tectum. **(A)** 7 dpf representative image of the *y304Et*(cfos:*Gal4*;UAS:*Kaede*) enhancer trap line used to obtain tectal cells. Optic tectum (white dashes), habenula (cyan arrows), epiphysis (yellow arrow), and olfactory system (magenta dashes) are indicated. The heart is not shown. **(B)** Experimental workflow for the collection and isolation of tectal cells. Heads from 7 dpf Kaede^+^ larvae were collected and enzymatically dissociated, followed by FACS to isolate fluorescent cells. After quality control, 14,704 single-cell libraries obtained by the 10X Genomics Chromium platform were used for downstream analysis. PCA and graph-based clustering were utilized to sort cells into clusters and identify candidate marker genes. Bioinformatic validation and spatial mapping was completed via fluorescent HCR RNA-FISH. **(C)** Uniform manifold approximation and projection (UMAP) showing cell populations identified via scRNA-seq analysis of 14,704 Kaede^+^ cells. Each point represents a single cell colored according to cluster identity as determined by unbiased graph-based clustering using the Louvain algorithm with multilevel refinement. UMAP embedding was used for visualization purposes only and not to define the clusters. The resulting two-dimensional spatial arrangement of clusters is a product of genetic similarity, as similar clusters are represented as closer together. **(D)** Candidate marker genes (columns) for each cluster (rows) were nominated by differential expression analysis wherein candidate genes must: (1) be expressed in at least 50% of cells within that cluster, and (2) show specificity to the cluster compared to all other cells by exhibiting ≥ 1 log2 fold-change in expression with at least 25% difference in gene presence. **(E)** Phylogram showing cluster relatedness based upon variable features defined by SCTransform and calculated within PCA space.

To obtain OT cells for scRNA-seq, we excised and collected heads from 7 dpf transgenic *y304Et*(cfos:*Gal4*;UAS:*Kaede*) larvae, an enhancer trap line which comprehensively labels the optic tecti, habenula, epiphysis, and heart; along with very sparse labeling in the olfactory bulbs (Figure 1A). Similar to previous scRNA-seq characterizations in zebrafish (Pandey et al., 2018; Lush et al., 2019; Kölsch et al., 2021), we found enzymatic dissociation with gentle trituration and fluorescence-activated cell sorting (FACS) at minimal flow rate to be an effective and robust method for preparing single cell suspensions for scRNA-seq (Figure 1B). To preserve transcriptional expression, we immediately methanol fixed cells post-FACS.

Using the droplet-based 10X Genomics Chromium platform, we sequenced RNA from 15,922 cells with an average of 1,882 transcripts and 729 genes recovered per cell. We filtered out low-quality cells by removing those with fewer than 500 UMI tags and 300 genes per cell, as well as cells with greater than 10% mitochondrial content. Following quality control, we scaled and normalized 14,704 cells and identified 33 distinct populations. Through differential expression analysis, we nominated sets of candidate marker genes that show specificity to their respective cluster. We used known marker genes for the heart, habenula, epiphysis, and olfactory system (Supplementary Table 1) to remove these populations from downstream analysis and designated the remaining 25 populations (13,320 cells) as putative tectal cells (Figure 1C). Although many clusters could be uniquely identified with a single marker gene (Figure 1D), in several instances clusters were best described using the top 5–10 differentially expressed genes (DEGs) (Supplementary Table 2). The identification of clusters in scRNA-seq data is typically accompanied by manual annotation of known cell types. However, prior to this study the transcriptomic knowledge of tectal cells has been limited, disallowing this method of cluster validation. To preclude over-clustering based on technical noise, we identified clusters based upon the presence of marker genes meeting our diagnostic criteria and conclude that at 7 dpf the larval zebrafish brain contains at least 25 transcriptionally distinct cell populations. Due to this conservative approach, it is possible that several OT clusters may include multiple cell types. Post-clustering, we determined the relatedness of OT clusters based upon top variable features identified by SCTransform during normalization and scaling (Figure 1E).

### Fluorescent *in situ* Hybridization Validates Bioinformatic Findings and Spatially Maps Populations of Interest

**FIGURE 2:**
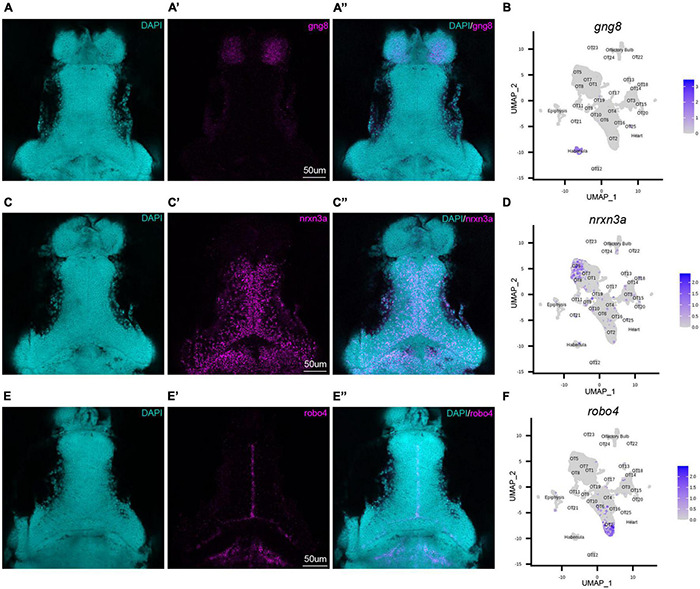
HCR RNA-FISH validates bioinformatic findings and spatially locates tectal populations. Hybridization chain reaction RNA-fluorescence *in situ* hybridization (HCR RNA-FISH) was performed using proprietary custom probe sets designed by Molecular Instruments to spatially map populations and validate bioinformatic exclusion of non-interest cells. **(A–A”,C–C”,E–E”)** Single z-slices of representative images showing expression of habenula (*gng8*) and OT (*nrxn3a*, *robo4*) markers in 7 dpf larval zebrafish. **(A–A”)** *gng8*^+^ cells are located in the habenula, validating *gng8* expression as a method for annotating habenular cells and spatially mapping 89% of habenula cells (Supplementary Table 2). **(B)** Feature plot showing *gng8* expression is restricted to the annotated habenula cluster. **(C–C”)** *nrxn3a*^+^ cells are primarily located medially within the periventricular layer, and spatially map 54% of OT5 (Supplementary Table 2). **(D)** Feature plot showing expression of *nrxn3a* is enriched in OT5 and to a lesser extent, OT8. **(E–E”)** *robo4*^+^ OT cells are located near the intratectal commissure, likely within the tectal proliferation zone, and spatially map 51% of OT2 cells (Supplementary Table 2). **(F)** Feature plot showing *robo4* expression is restricted to OT2. All HCR RNA-FISH panels include DAPI staining as a cellular reference.

To validate our transcriptomic and clustering data, we performed hybridization chain reaction RNA fluorescence *in situ* hybridization (HCR RNA-FISH) using custom probes for *gng8*, *nrxn3a*, and *robo4* (Molecular Instruments, Los Angeles, CA). *gng8*, a well-known habenula marker, was used among other determinant genes to annotate and remove habenular populations (Supplementary Figure 4A). HCR RNA-FISH targeting of *gng8* confirmed that its expression is limited to the habenula (Figures 2A–A”), validating the clustering algorithm (Figure 2B) and our method of cell exclusion. In addition to bioinformatic validation, we used HCR RNA-FISH labeling to spatially map previously uncharacterized populations. HCR RNA-FISH of OT5 marker *nrxn3a* (Figures 1D, 2C–C”), confirmed expression is found in the OT. Moreover, it showed that *nrxn3a*^+^ cells are primarily found deep within the periventricular layer, with most located near the intratectal commissure that separates each tecti (Figure 2C’). Additionally, it is important to note that although *nrxn3a* is expressed higher within OT5 than other populations, it is not exclusive to it (Figures 1D, 2D); thus, we anticipate HCR RNA-FISH labeling includes a portion of OT8 cells as well. Lastly, we performed HCR RNA-FISH of *robo4*, a marker that labels 51% of the OT2 cluster (Supplementary Table 2), and found its expression to be limited to OT2 cells in the tectal proliferation zone (Figures 2E–F). Taken together, HCR RNA-FISH validated our bioinformatic method of excluding cells of non-interest and shows its capability for high resolution labeling and spatial mapping of OT populations of interest.

### The Larval Optic Tectum Contains Mature and Developing Neuronal Populations as Well as Several Glial Types

**FIGURE 3:**
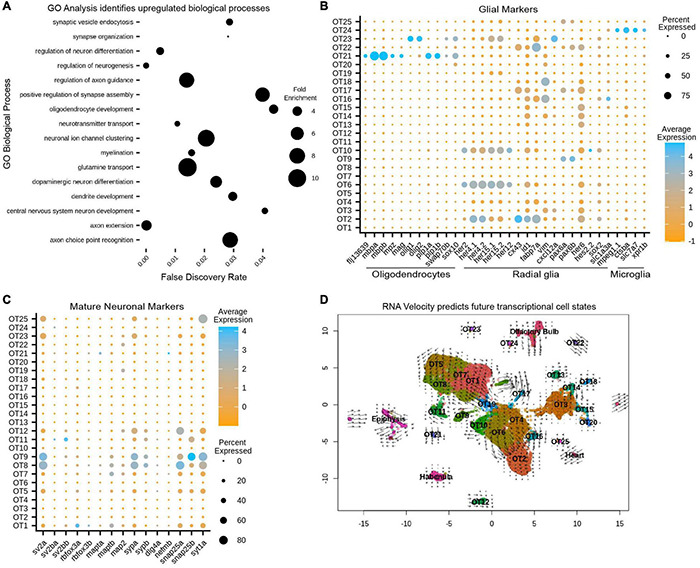
Gene ontology (GO) and RNA velocity analysis reveals upregulated biological processes and predict future transcriptional cell states. **(A)** Upregulated processes in all 13,320 putative tectal cells. Whole dataset GO analysis was performed on all significant (*p* < 0.05) differentially expressed genes (see also Supplementary Table 2). Processes of note include regulation of axon guidance, positive regulation of synapse assembly, glutamine transport, and oligodendrocyte development, suggesting the presence of immature/mature neuronal populations and glial cells (see also Supplementary Tables 3, 6). **(B)** Cluster analysis of glial markers identifies OT21/OT23 as oligodendrocytes; radial glial markers are found in various populations including OT6 and OT10; OT24 is likely microglial (see also Supplementary Table 5). **(C)** Cluster analysis of manually curated neuronal markers shows several mature populations including OT8 and OT9 (see also Supplementary Table 4). **(D)** RNA velocity with original UMAP cluster embeddings. Shorter arrows or dots connote more mature cells. Arrows are vectors where direction indicates the predicted transcriptional state and magnitude indicates the degree of differentiation. BAM files from two temporal replicates were used to generate a matrix containing spliced:unspliced count ratios for each gene, where higher spliced counts indicate a more processed form of the gene and lower spliced counts indicate newly “born” genes.

As a preliminary survey, we performed whole-dataset Gene Ontology (GO) analysis to identify which biological processes are upregulated in the larval optic tectum at 7 dpf. Although GO analysis is often used to explore gene expression differences between conditions; it can be a valuable exploratory tool when approaching characterization of an unknown structure or developmental stage. We implemented the MAST test to identify 2,222 DEGs that were used as input for GO analysis (Supplementary Table 2) and found upregulated processes such as *myelination* and *synaptic vesicle endocytosis*, indicating the presence of glial cells and mature neurons, (respectively). We also found processes such as *axon guidance* and *neuron differentiation*, indicating neuronal populations may still be developing as these processes would not be enriched in fully mature neurons (Figure 3A and Supplementary Table 3). However, whole-dataset GO analysis is limited in scope as a characterization tool as (1) it cannot determine which populations are contributing to particular processes, and (2) does not indicate whether evidence of developing neuronal populations is due to the ongoing neurogenesis seen throughout the zebrafish life, or is simply indicative of a structure that has not yet reached maturity. Conversely, conducting individual cluster analysis can misrepresent data during the overrepresentation comparison due to large discrepancies in cluster size. For these reasons, we used whole-dataset GO analysis exclusively as an initial screening tool to guide expression analysis and not as a strict determination of OT developmental state.

Within our 25 putative OT populations, we characterized OT21 and OT23 clusters as oligodendrocyte populations, demonstrated by their enriched expression for *mbpa/b* and *olig1/olig2*, (respectively), compared to all other OT cells (Figure 3B). We next identified OT2, OT6, and OT10 (among others) to be enriched for radial glial markers such as *her4.1*, *her4.2*, and *fabp7a* (Figure 3B). In addition, OT2 shows unique differential expression of *mfeg8a*, *id1*, and *cx43* (Figures 1D, 3B) which have been previously described as quiescent radial glial markers (Lange et al., 2020). Lastly, we identified OT24 as microglial due to expression of *mpeg1.1*, *slc7a7*, and *ctsba* (Figure 3C), the latter a lysosomal gene found to be enriched in tectal populations of microglia (Silva et al., 2021; see Supplementary Table 5 for all glial markers).

We also used marker genes for neurons (Supplementary Table 4) to identify OT5, OT8, and OT9, among others, as mature post-mitotic neurons actively involved in neurotransmission (Figure 3C). In contrast to these mature populations, we found clusters such as OT6 and OT10 show upregulated neurogenesis genes (Supplementary Figure 7), suggesting they are likely contributing to the enriched neuronal development processes seen previously in GO analysis (Figure 3A).

In conclusion, the 7 dpf larval optic tectum contains at least three non-neuronal cell types and 22 neuronal populations. Of these, we consider 7 to be mature and 15 to be immature. However, neurodevelopment is a process, and some of these immature populations are further along their developmental trajectory than others. We recognize that the characterization of mature vs. immature populations, particularly at a single-time point, may be subjective between researchers. A summary of our classifications of OT cell type, developmental stage, and neuronal profile (if applicable) is found in Table 1.

**TABLE 1:** Summary of 25 transcriptionally distinct tectal cell populations.

Population	Designation	Neuronal profile	Marker genes	Potential links to previous studies
OT1	Late—term developing neuronal	GLU; synapsing with GAB	ebf3a.1*, lhx9, barhl2, pou4f2, lmo3	
OT2	Intermediate neural progenitor	GAB°, GLY; synapsing with GLU	mfge8a*, cx43, hspb15, slc1a2b, slc4a4a	
OT3	Developing neuronal	Potential synapsing with GLU	col1a1a*, thbs4b, tat, col1a1a, col6a2	*dlx5a*^+^, three known morphologies (Robles et al., 2011)
OT4	Late—term developing neuronal	Synapsing with GLU	npm1a*, cnn2, cad, hsd17b12a, dkc1	
OT5	Mature neuronal	GAB; synapsing with GLU	nrxn3a*, sox14, tal1, gata3, zfpm2b	
OT6	Developing neuronal	Synapsing with GLU	her15.1*, si:ch73-21g5.7, abhd6a, her12, her2	
OT7	Mature neuronal	GAB°, GLU; synapsing with GAB, GLU	tubb5*, neurod1, znf536, nfia, r3hdm1	*dlx5a*^+^/*dlx6a*^+^, three known morphologies (Robles et al., 2011)
OT8	Mature neuronal	GAB°, GLU, GLY; synapsing with GAB, GLU, GLY	elavl4*, npas4a, gad1b, adcyap1r1a.1, slc12a5b	*dlx5a*^+^/*dlx6a*^+^, three known morphologies (Robles et al., 2011)
OT9	Mature neuronal	GAB°, GLU; synapsing with GLU, GLY, CHO	tkta*, rbpms2a, pax6b, rbpms2b, SYNPR	
OT10	Developing neuronal	Synapsing with GLU	hmgb2a*, cdk1, cenpf, tacc3, aspm	
OT11	Late—term developing neuronal	Synapsing with GAB, GLU	syt5b*, cabp2a, vsx1, cabp5b, lrit1a	
OT12	Mature neuronal	GLU°, CHO; synapsing with GAB, GLU, CHO	si:dkey-117i10.1*, prkcq, kiss1, gpr139, ppp1r14ab	
OT13	Developing neuronal	Potential synapsing with GLU	dusp5*, f8, ptprb, egfl7, tie1	*id2b*^+^, three known morphologies (DeMarco et al., 2020)
OT14	Developing neuronal	Potential synapsing with GLU	mmel1*, gstm.3, myrf, tmem98, tmem88b	
OT15	Developing neuronal	Potential synapsing with GLU	matn4*, otos, col9a1a, col2a1a, cpxm1a	
OT16	Developing neuronal	Potential synapsing with GLU	foxj1a*, ldlrad2, tbata, fabp7b, rab36	*id2b*^+^, three known morphologies (DeMarco et al., 2020)
OT17	Mature neuronal	GLY; synapsing with GLU, DOP	kcnk2a*, slc45a2, col9a1b, zgc:114181, wfdc1	
OT18	Developing neuronal	Potential synapsing with GLU	cfd*, elnb, myh11a, si:dkey-57k2.6, acta2	
OT19	Developing neuronal	Potential synapsing with GLU	tmsb*, dlb, gadd45gb.1, insm1a, pou2f2a.1	*dlx5a*^+^/*dlx6a*^+^, three known morphologies (Robles et al., 2011)
OT20	Late—term developing neuronal	Potential synapsing with GLU	fgfbp2b*, mia, tgm2l, chad, snorc	
OT21	Oligodendrocytes	–	mbpa*, mpz, cd59, cldnk, plp1b	
OT22	Developing neuronal	CHO°, GAB; synapsing with GLU	cavin2a*, hyal6, ca14, lmo7b, rlbp1a	
OT23	Mixed population of oligodendrocytes and mature neurons	GAB; synapsing with GLU	bcan*, olig1, aplnrb, kcnj11l.1, olig2	
OT24	Microglia	–	pfn1*, zgc:173915, ccl34b.1, havcr1, fcer1g	*mpeg1.1*^+^, *ctsba*^+^, ameboid morphology (Rossi et al., 2015; Silva et al., 2021)
OT25	Mature neuronal	GAB°, GLU; synapsing with GLU, GLY	si:ch211-48m9.1.1*, si:ch211-276i12.4, prkar2ab, CABZ01114053.1, rprmb	*dlx6a*^+^, three known morphologies (Robles et al., 2011)

*A brief overview of transcriptomic findings from targeted scRNA-seq of the larval zebrafish optic tectum.*

*GLU, glutamatergic; GLY, glycinergic; GAB, GABAergic; CHO, cholinergic; DOP, dopaminergic.*

*Population marker gene.

*°Primary neurotransmitter identity.*

### Trajectory Analysis via RNA Velocity Can Predict Future Transcriptional Cell States

**FIGURE 4:**
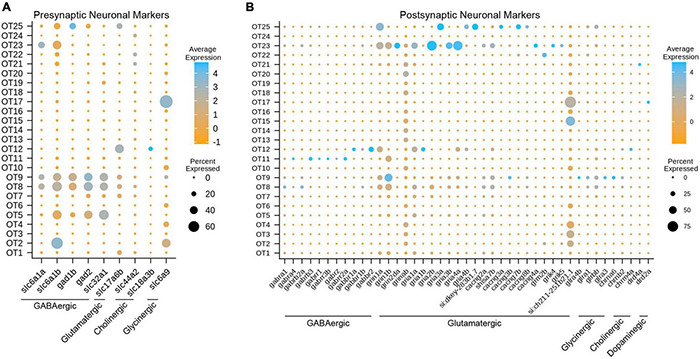
Gene expression profiles characterize the neuronal profiles of OT cells. **(A)** Select presynaptic markers identify mature inhibitory (GABAergic, glycinergic, cholinergic) and excitatory (glutamatergic) tectal populations. **(B)** Select postsynaptic markers identify the potential synaptic partners of tectal neurons by exploring genes required for various neurotransmitter receptors. All markers were manually curated based on functional designation on the ZFIN database (https://zfin.org/); see Supplementary Table 7 for all pre- and postsynaptic markers.

To characterize the current transcriptional states of OT clusters and predict their future states, we performed RNA velocity analysis, which uses the ratio of unspliced to spliced gene transcripts of a gene (or proportion of intron retention), to estimate the directionality and magnitude of transcriptional changes on the timescale of hours (La Manno et al., 2018). Conceptually, a higher ratio of unspliced:spliced indicates “newer” genes that are actively upregulated, while a lower ratio indicates mature, highly processed transcripts. This distinction is what enables RNA velocity analysis to infer current and future transcriptional cell states for each cluster. Thus, we calculated and displayed velocity estimates on our original UMAP to visualize predicted transcriptional cell states (Figure 3D). Superimposed dots indicate mature, terminal populations, while arrows can be interpreted as vectors, where the direction and magnitude indicate the predicted future transcriptional state. Notably, clusters OT8, OT24, and OT25 appear to be terminally differentiated while OT6 and OT16 are actively differentiating, evidenced by superimposed arrows over these clusters pointing to OT2 (Figure 3B). This corroborates our previous assessment that these populations are likely developing neurons and predicts that if development were to proceed normally, they may eventually transcriptionally resemble OT2. It is important to clarify that RNA velocity does not predict cell identity, and we cannot conclude that OT6 will develop into OT2. However, it may eventually share transcriptional similarities such as a GABAergic identity (Figure 4A), but all GABAergic neurons are not identical in morphology, circuitry, or function. Similarly, we do not anticipate all OT2 cells to be mature neurons due to an upregulation of radial glial genes and neurogenesis markers (Figure 3C and Supplementary Figure 7). Thus, we use RNA velocity as a tool to characterize current developmental states and predict future transcriptional cell states, but not to perform lineage tracing to determine future cell identities. Taken together, our RNA velocity results corroborate immature and mature population findings and build upon those observations by predicting the future transcriptional state of differentiating populations.

### Most Mature Tectal Neurons Send Inhibitory Signals and Receive Excitatory Input

To determine the neuronal profiles of mature tectal neurons, we used pre- and postsynaptic marker genes for different neurotransmitter types (Supplementary Table 7). We determined that at 7 dpf, the zebrafish optic tectum contains a strong presence of mature GABAergic neurons, and to a lesser extent, glutamatergic and glycinergic neurons (Figure 4A). This is consistent with previous studies describing GABAergic neurons as the majority population (Robles et al., 2011; Barker et al., 2020; DeMarco et al., 2020). While dopaminergic and serotonergic populations were not observed, GABAergic population OT9 is of particular interest, as those cells express glutamatergic, glycinergic, and cholinergic receptor-building genes (Figure 4B and Table 1); suggesting that this population receives input from various neuronal types. Interestingly, we found OT17 to highly express *slc6a9* (GLYT1), which encodes a transporter responsible for presynaptic re-uptake of the inhibitory neurotransmitter glycine. Previous studies have described tectal GLYT1 expression at 24 hpf, while both GLYT1 and GLYT2 are found in the hindbrain and spinal cord at later stages in zebrafish (Cui et al., 2005). Overall, we found major GABAergic populations such as OT8 AND OT9 to differentially express glutamatergic receptor genes, indicating their main synaptic partners are excitatory.

### Optic Tectum Populations Differentially Express Transcription Factors

**FIGURE 5:**
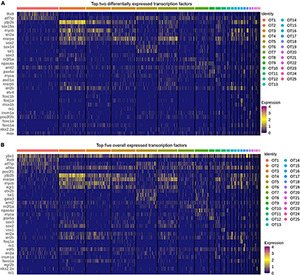
Top transcription factors in the larval zebrafish OT. **(A)** The top two differentially expressed transcription factors in each OT population as compared to all other cells. **(B)** Top five expressed transcription factors in each population. **(All)** Duplicates of top factors are allowed but displayed only once. Factors were considered differentially expressed for a particular cluster if they were expressed > 0.25 log2 fold change above all other cells and are present > 25% of cells within the cluster. Factors were ordered in decreasing expression (log2fc) level and the top two or five were selected for visualization; see Supplementary Table 8 for all transcription factors.

Often overlooked in scRNA-seq analysis, transcription factors are the master regulators of gene expression and offer valuable cell identity information concerning the establishment and maintenance of cell fate. To explore these within OT populations, we obtained a list of all reviewed zebrafish transcription factors (310 at time of analysis) from The UniProt Consortium (2021). Of these 310 reviewed factors, 257 were present in the OT to some degree, and only 42 of the 257 were considered differentially expressed (>0.25 log2fc, > 25% of cluster cells). We identified the top two differentially expressed transcription factors (Figure 5A) within each cluster; meaning they are expressed more highly within that population compared to all other cells, not that they are unique to that population alone. Of particular interest is the strong upregulation of *jdp2b* in OT2 (Figure 5A). *jdp2b* protein represses AP-1, a separate transcription factor that functions in cell proliferation and differentiation (Ku et al., 2021), indicating OT2 may be nearing its developmental end. This is consistent with previous conclusions identifying OT2 as a developmentally heterogeneous population, with indications of both developing (Figure 3C and Supplementary Figure 7), and mature features (Figures 4A,B).

In contrast to the relative specificity of *jdp2b* to OT2, we note that each population does not show cluster-specific transcription factor expression. Although these duplicate factors are displayed only once in each heatmap, many cell populations express the same or similar transcription factors. However, it is highly unlikely that these factors modulate gene expression in identical manners across the entire optic tectum. Rather it is the unique combination of multiple factors, coupled with developmental stage and tissue, which work together to establish and maintain that cell’s identity. To include this likelihood in our characterizations, we not only looked at differentially expressed transcription factors, but also top expressed (in terms of percentage of expressing cells) in each cluster. We found several factors, *ybx1* and *atf4a*, to be fairly ubiquitous across all OT populations (Figure 5B) despite the molecular heterogeneity of OT cells. In this regard, *ybx1* and *atf4a* pose an interesting avenue for further investigations regarding regulation of tectal cell identity.

### Tectal Cell Populations Show Differential Expression of Genes Implicated in Autism Spectrum Disorder

**FIGURE 6:**
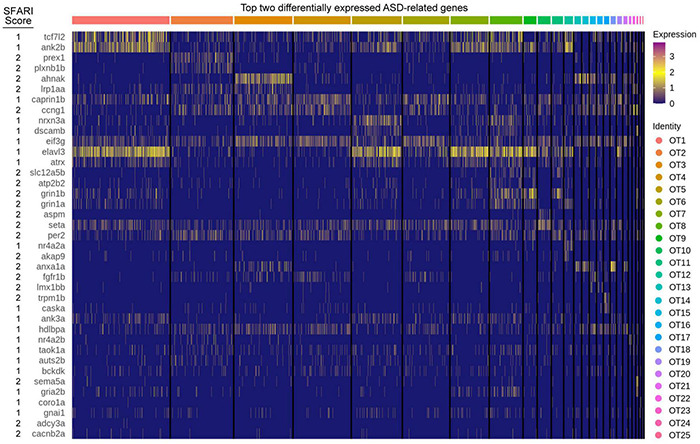
ASD implicated genes are differentially expressed in the larval zebrafish OT. The top two differentially expressed genes implicated in ASD pathogenesis, duplicates are allowed but only displayed once. Score: Indicates ASD association score conferred by SFARI Gene. See Supplementary Table 8 for all ASD implicated genes and their respective scores.

The optic tectum, like its mammalian counterpart, the superior colliculus (SC), is a sensory processing hub that receives multimodal stimuli and is responsible for eliciting appropriate behavioral responses (Stein and Stanford, 2013). Recently, the SC has received attention for a proposed role in autism spectrum disorder (ASD) pathogenesis due the essential role it plays in sensory perception (Jure, 2019). Considering the homologous nature of the optic tectum, we investigated the tectal expression of 316 genes (407 zebrafish orthologs and paralogs) implicated in ASD pathogenesis (Supplementary Table 8). Of those, 348 were present in the OT to some degree, while 80 were considered differentially expressed (> 0.25 log2fc, > 25% of cluster cells). We found several genes, such as *seta* and *eif3g*, to be expressed consistently across all clusters, while others showed greater cluster specificity, such as *prex1* (OT2), *nrxn3a* (OT5), *aspm* (OT10), and *nr4a2a* (OT12) (Figure 6). This population-specific differential expression of ASD-implicated genes highlights the potential for investigations into the hypothesized link between the superior colliculus (or optic tectum) and ASD pathogenesis.

## Discussion

To compliment current tectal characterization efforts, we describe the transcriptomic profiles for 25 molecularly distinct tectal cell populations including novel marker genes, neurotransmitter identities, potential synaptic partners, transcription factor expression, and developmental predictions.

### Developmental State of the Optic Tectum at 7 Days-Post-Fertilization

Previous functional studies have shown escape, prey detection, and orientation behaviors are present in larval zebrafish (Fero et al., 2011), indicating that at 7 dpf the OT has some level of functional maturity. Our data supports this through the identification of several mature neuronal populations. However, we also see several clusters that differentially express neurogenesis genes as well as markers for radial glia. In this context, it is important to note that as these cells give rise to neurons, expression of their markers may denote immature neurons along with true mature radial glia. Mammalian radial glia disappear rapidly after birth, but are present in adult zebrafish which, like all teleosts, undergo widespread neurogenesis throughout their life (Schmidt et al., 2013). In the adult OT, proliferating cells in a PVL neurogenic niche have been confirmed through bromodeoxyuridine (BrdU) labeling to differentiate into multiple cell lineages, confirming the OT is a site of ongoing neurogenesis (Ito et al., 2010; Schmidt et al., 2013). However, in contrast to stem cell niches in adult mammalian brains, these proliferating cells do not co-express neural progenitor and radial glia markers (Ito et al., 2010). The populations we have classified as immature express both types of markers, suggesting they are not the same proliferating cells seen in the adult OT. Although radial glia expressing neural progenitor markers are also present in the adult OT, it is unlikely that all immature clusters represent true radial glial cells. Thus, while it is difficult to differentiate between continued larval development and ongoing neurogenesis, we hypothesize these immature populations likely indicate that at 7 dpf the OT is still finetuning development. However, we acknowledge that development and ongoing neurogenesis are not mutually exclusive and likely overlap one another; further lineage tracing or time-series sequencing of OT populations may elucidate this.

### Neuronal Cell Types in the Larval Optic Tectum

A wealth of data exists concerning tectal cell characterization; however, many studies are conducted using enhancer trap lines and as a result, have limited gene expression information despite excellent morphological characterization. Although we did not determine the morphologies of each population, we demonstrate the ability to connect our transcriptomic data with previous work by highlighting several highly influential studies that characterized various tectal cell types (Table 1).

In 2011, Robles et al. (2011) utilized a transgenic line labeling *dlx5a*/*dlx6a* positive neurons in the larval zebrafish optic tectum, identifying three morphological classes: GABAergic non-stratified periventricular interneurons (nsPVINs), glutamatergic bi-stratified periventricular interneurons (bsPVINs), and GABAergic periventricular projection neurons (PVPNs). In our data, 5 populations express either *dlx5a*, *dlx6a*, or both. It is possible that nsPVIN and PVPN morphologies denote cells within GABAergic OT7 or OT8 clusters, which express both *dlx5a*/*dlx6a*. bsPVIN morphologies may be found in OT25, which expresses *dlx6a* and contains glutamatergic neurons. However, as many neuronal populations are still immature at 7 dpf, these morphologies may also be found in populations such as OT19, which expresses both *dlx5a*/*dlx6a* but is still developing.

A second study conducted in 2020 used the *id2b:Gal4* transgene to label a subset of tectal neurons in larval zebrafish (DeMarco et al., 2020). Similar to the Robles study, genetic mosaic labeling of single neurons led to the identification of three tectal morphological types (in descending proportion): pyramidal neurons (PyrNs), projection neurons that project to the torus longitudinalis (TLPNs), and a second projection neuron type that projects to the tegmentum (TGPNs). Surprisingly, most *id2b*(*EGFP*)^+^ cells appear to be cholinergic (likely PyrNs), while approximately one-third are glutamatergic (likely TLPNs), and only 10% are GABAergic (likely TGPNs) (DeMarco et al., 2020). We found few cholinergic neurons; however, two developing populations without a known neurotransmitter identity show expression of *id2b*, thus we hypothesize these populations may have PyrN, TLPN, or TGPN morphologies.

Superficial interneurons (SINs) have also been described as primarily GABAergic and receiving glutamatergic input directly from retinal ganglion cells (Barker et al., 2020). Of the eight populations in our data that appear to contain mature neurons, four are GABAergic and synapse with glutamatergic neurons. These populations likely include SINs; however, we also identified a glutamatergic population that receives both GABAergic (inhibitory) and glutamatergic (excitatory) input. Thus, populations that demonstrate excitatory-inhibitory connections likely include SINs, but our data suggests these connections occur within the microcircuitry of the tectum as well.

Lastly, we identified a single glycinergic population, OT17, through enrichment of *slc6a9*/GLYT1 which encodes a glutamine transporter responsible for glycine reuptake into the presynaptic cell. GLYT1 may also be expressed by astrocytes serving to regulate neurotransmission, and although previous work found GLYT1 expression in the tectum through *in situ* hybridization at 24 hpf (Cui et al., 2005), it was unclear if these were neurons or glia. Glial cells which share similar functions and characteristics to mammalian astrocytes have been previously described in zebrafish. These astrocyte-like glial cells have been shown to modulate neurotransmission, react to injury, and express similar molecular markers as their mammalian counterparts (Grupp et al., 2010; Grandel and Brand, 2013; Lyons and Talbot, 2015). In larval zebrafish, tectal and cerebellar astrocyte-like cells play a role in the initiation and propagation of generalized epileptic seizures (Diaz Verdugo et al., 2019), while in the hindbrain they regulate behavioral passivity following failed swim attempts (Mu et al., 2019). However, they were not fully characterized until 2020 when Chen et al. (2020) identified astrocyte-like cells with enrichment of glutamine synthase (*glula*) and glutamate aspartate transporter 1 (*slc1a3b*/GLAST); along with *fgfr3*, and *fgfr4*, signaling factors required for proper astrocyte growth and function in *Drosophila* (Muñoz-Ballester et al., 2021). However, OT17 does not highly express any genes described by Chen et al. (2020) (data not shown), rather it shows expression of glutamatergic and dopaminergic receptor genes. Therefore, we believe it likely represents a true glycinergic neuronal population rather than a modulatory astrocyte-like cell. Although previous work has concluded a lack of tectal glycinergic neurons in zebrafish (Barreiro-Iglesias et al., 2013), this was investigated via a transgenic line where GFP is driven by the GLYT2 promoter. This is supported by our data, as we see a lack of GLYT2 expression across OT populations. However, GLYT2 may not be sufficient to identify all glycinergic neurons, as a recent transgenic line that uses a GLYT1 BAC to drive GFP expression marks inhibitory amacrine neurons within the zebrafish retina (Wang et al., 2020). Thus, GLYT2 expression cannot be used as an exclusive marker of glycinergic neurons. In fact, our study presents evidence for GLYT1^+^ glycinergic neurons within the optic tectum and suggests that characterization via GLYT1 and not GLYT2 may provide insight into the development and function of glycinergic neurons in the OT.

### Non-neuronal Cells of the Larval Optic Tectum

As previously discussed, true radial glia can be difficult to identify as many markers are simply genes involved in neurogenesis. At 5 dpf, the larval OT contains a proliferation zone that functions as a post-embryonic neurogenic niche, giving rise to neurons through *her4*^+^ transient radial glia cells (Boulanger-Weill and Sumbre, 2019). Among the *her4*^+^ populations we identified, OT2 shows an enrichment of quiescent radial glial markers described by Lange et al. (2020) during transcriptomic characterization of ongoing neurogenesis in the adult zebrafish brain. However, designation of OT2 as quiescent radial glia may be preemptive, as our scRNA-seq data shows exclusive OT2 expression of *robo4*, which is typically found in neurons. Furthermore, our HCR RNA-FISH localizes *robo4*^+^ OT2 cells to the tectal proliferation zone. In mice, *robo4* is expressed in newly born cortical neurons, and its knockdown results in severe radial migration defects in layers II/III of the developing cortex (Zheng et al., 2012). Thus, concurrent expression of *robo4* and quiescent radial glial markers indicate that OT2 may represent an intermediate between these two populations. OT2 also shows low expression levels of *robo1* and *robo2.* In a study by Cárdenas et al. (2018) and Gonda et al. (2020) low expression levels of these genes in neocortical progenitor cells were found to maintain indirect neurogenesis in the neocortex. This further supports the hypothesis that OT2 is somewhere along a neurogenic path and may represent an intermediate progenitor population transitioning from a quiescent radial glial state into newly born neurons.

Three populations containing non-neuronal cells were identified in our data. Two appear to represent oligodendrocytes, which have been previously identified in the deeper portions of the tectal PVL (Ito et al., 2010). A small population of microglia with an amoeboid morphology has been recently described to localize near neurogenic regions and express the myeloid reporter gene *mpeg1.1, as* well as lysosomal genes *ctsla* and *ctsba* (Silva et al., 2021). We found a single small population to express *mpeg1.1*, and *ctsba*; as well as *slc7a7*, which functions in microglia development (Rossi et al., 2015). From this evidence, we conclude our third non-neuronal cell type to be microglia.

### The Optic Tectum May Provide a Useful Model for Autism Pathogenesis Studies

A potential link between the SC and autism spectrum disorder (ASD) has been proposed as congenitally blind children are diagnosed with ASD at a higher rate than those born with no vision impairments (Jure et al., 2016; Jure, 2019). As sensory processing impairments typically present in ASD, a structure such as the SC that receives and interprets sensory stimuli poses an interesting avenue for ASD investigations. Utilizing a homologous structure that is highly amenable to genetic manipulations, such as the zebrafish OT, may inform a potential link between the SC and ASD in humans.

Despite clinical heterogeneity and a largely unknown etiology, genetics believed to be a significant contributing factor to ASD. The highly diverse neurexin family of presynaptic cell adhesion molecules is one of the few gene families where every member (NRXN1/NRXN2/NRXN3) is highly implicated in ASD (Banerjee-Basu and Packer, 2010; Cao and Tabuchi, 2017; Tromp et al., 2021). Diversity can be attributed to alternative promoters generating alpha (long) and beta (short) isoforms, which are then subject to extensive alternative splicing (Ullrich et al., 1995). Structure is consistent across family members, and alpha/beta isoforms share an intracellular domain as well as one extracellular binding domain (Cao and Tabuchi, 2017). Although neurexin function is not fully understood, they are hypothesized to interact with postsynaptic neuroligin proteins to induce synapse formation (Ullrich et al., 1995).

A genome duplication after mammalian and teleost lineages diverged resulted in two zebrafish orthologs for many human genes (Amores et al., 1998; Postlethwait et al., 1998), including neurexins. Six neurexin genes (*nrxn1a*/*b*, *nrxn2a*/*b*, and *nrxn3a*/*b)* have been identified but under characterized in zebrafish, although all were shown to be required for synaptogenesis and alpha-*nrxn2a* deficiency was shown to result in increased anxiety (Rissone et al., 2007; Shah et al., 2015; Koh et al., 2020). We found the main ortholog of NRXN3 (*nrxn3a*) is differentially expressed within specific populations in the larval OT (Figures 2C–C”, 6); thus, investigations into a potential SC/OT—ASD link may benefit through the characterizations of neurexins in the OT.

### Transcriptomic Characterization of the Larval Optic Tectum Facilitates Future Studies

The amount of information connecting gene expression and tectal cell morphologies is less than ideal; by providing the transcriptomic profiles of tectal cells, we provide the genetic information necessary for future studies to target individual populations and further these associations. This can allow for a variety of experiments such as (1) the use of a population marker to drive reporter gene or calcium indicator expression to observe morphologies or conduct functional assays; or (2) use of previous transgenic lines to drive knock-out or knock-down of marker genes or other highly expressed genes, such as unique transcription factors. Along with providing additional genetic information for future studies, we provide strong evidence that the OT is still undergoing significant developmental fine-tuning. As many functional and molecular studies are typically conducted in relatively young larvae (3–10 dpf), extending experimental windows to include older larvae/adults may benefit functional OT understanding as a whole. We conclude that although the goal of complete characterization will require still more work, the molecular profiles we have compiled of 25 tectal populations will be a valuable resource in relating transcriptional identity with functional identity.

## Data Availability Statement

The datasets presented in this study can be found in online repositories. The names of the repository/repositories and accession number(s) can be found below: https://www.ncbi.nlm.nih.gov/sra, PRJNA779441.

## Ethics Statement

The animal study was reviewed and approved by the Institutional Animal Care and Use Committee (IACUC) at Brigham Young University.

## Author Contributions

AM, AB, and AP conceived and performed experiments. AS conceived and provided guidance on experiments. AM performed all bioinformatic analyses. AM, AB, AP, and AS wrote the article. JH and BP provided sequencing and bioinformatic counsel. All authors contributed to article review and revision.

## Conflict of Interest

The authors declare that the research was conducted in the absence of any commercial or financial relationships that could be construed as a potential conflict of interest.

## Publisher’s Note

All claims expressed in this article are solely those of the authors and do not necessarily represent those of their affiliated organizations, or those of the publisher, the editors and the reviewers. Any product that may be evaluated in this article, or claim that may be made by its manufacturer, is not guaranteed or endorsed by the publisher.

## Footnotes

1
https://support.10xgenomics.com/single-cell-gene-expression/software/overview/welcome

2
https://satijalab.org

3
https://zfin.org/

4
www.pantherdb.org

5
https://velocyto.org/velocyto.py/

6
https://molecularinstruments.com

7
https://ncbi.nlm.nih.gov/gene/

8
https://uswest.ensembl.org/

9
https://molecularinstruments.com/hcr-rnafish-protocols

## Supplementary Material

Supplementary Figure 1 Sorting of kaede^+^ cells for single-cell RNA sequencing, related to methods.

Supplementary Figure 2 Quality control for cell cycle regression, related to methods.

Supplementary Figure 3 Quality control check for batch effect, related to methods.

Supplementary Figure 4 Annotation of habenula and heart cells, related to methods and Figure 1.

Supplementary Figure 5 Annotation of epiphysis and olfactory bulb cells, related to Figure 1 and methods.

Supplementary Figure 6 Detection of *kaede* and genes proximal to *Gal4* insertion, related to methods.

Supplementary Figure 7 Top five unique neurogenesis genes, related to Figure 3 and Table 1.

Supplementary Table 1 Markers for non-tectal annotation, related to Figures 1, 2, and methods.

Supplementary Table 2 All differentially expressed genes identified by MAST algorithm, related to Figures 1, 3, and methods.

Supplementary Table 3 Analysis summary of gene ontology PANTHER overrepresentation test, related to Figure 3 and methods.

Supplementary Table 4 Mature neuronal markers, related to Figures 3, 4, and methods.

Supplementary Table 5 Glial markers, related to Figure 3 and methods.

Supplementary Table 6 Genes associated with “neurogenesis” gene ontology term, related to Figure 3 and Table 1.

Supplementary Table 7 Pre- and postsynaptic neurotransmitter markers, related to Figure 4 and Table 1, and methods.

Supplementary Table 8 SFARI category 1–2 ASD genes and reviewed UniProt transcription factors, related to Figures 5, 6, and methods.
