# Supplementary material for: Single-Cell RNA Sequencing Characterizes the Molecular Heterogeneity of the Larval Zebrafish Optic Tectum
Source: Front Mol Neurosci. 2022 Feb 10;15:818007. doi: 10.3389/fnmol.2022.818007 (PMC8869500; doi:10.3389/fnmol.2022.818007)
Supplement: Supplementary Figure 1 — Sorting of kaede+ cells for single-cell RNA sequencing, related to methods. [file Data_Sheet_1.pdf]

I

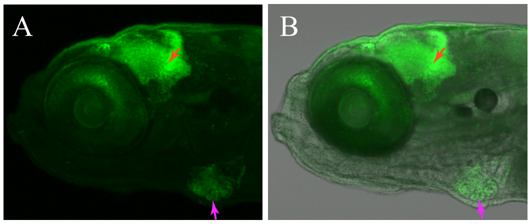

II

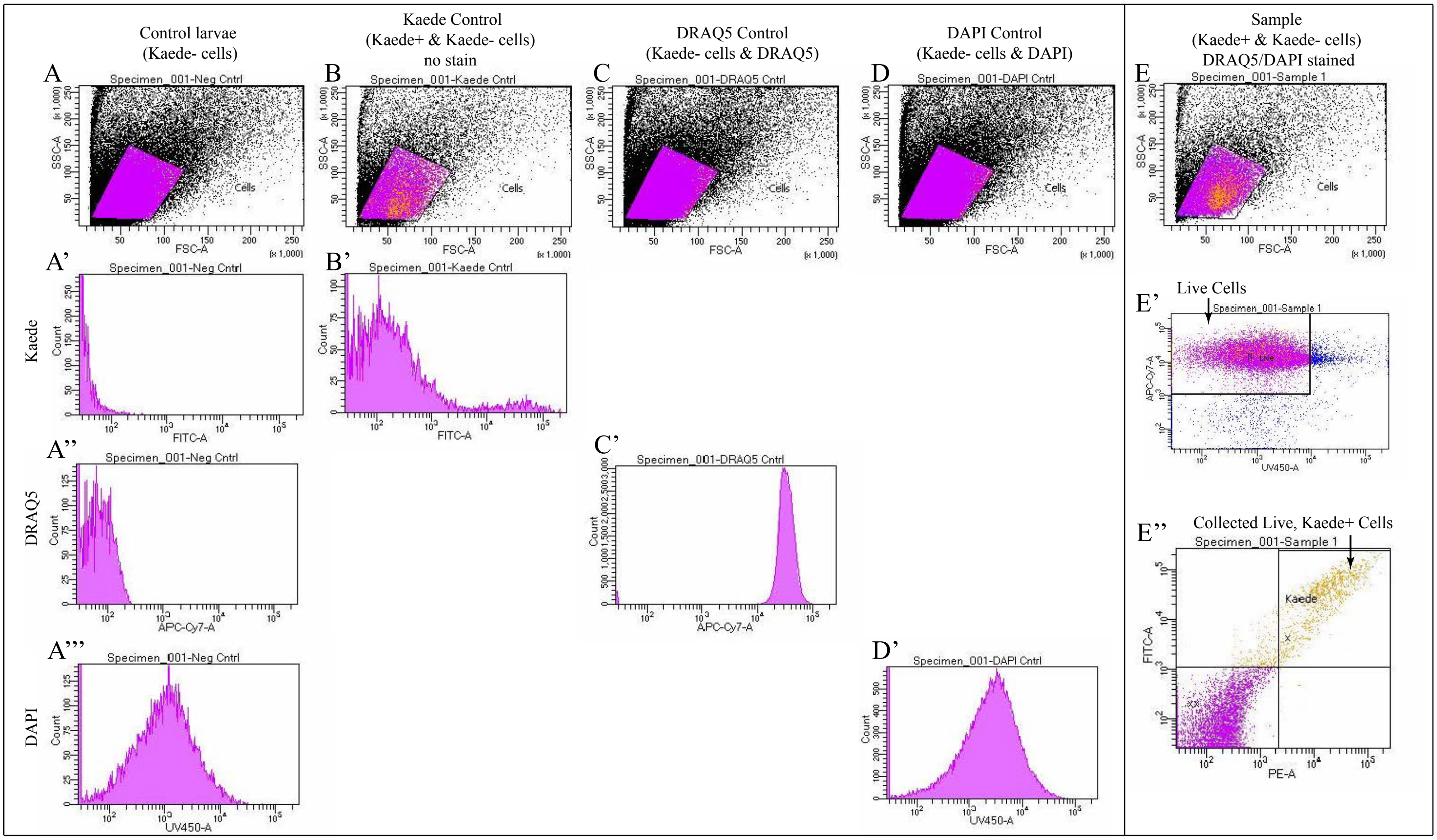

Supplemental Figure 1. Sorting of Kaede⁺ Cells for Single-Cell RNA Sequencing, Related to Methods.

I) The y304 enhancer trap line labels structures including the optic tectum, habenula, epiphysis and heart. A-B) Lateral view of a 7dpf larva showing Kaede expression in the optic tectum (red arrow) and heart (magenta arrow). B) Overlay of the fluorescent image in A on the transmitted image of the same larva. Prior to imaging larva was PFA fixed and stored in methanol, which may result in low levels of autofluorescence.

II) We performed fluorescent activated cell sorting (FACS) to collect Kaede^{+} cells from the Y304 enhancer trap line. Heads from 7dpf larval zebrafish were enzymatically dissociated and sorted using the FACS Aria Fusion Cell Sorter. Cells with a particular size and complexity were selected for sorting (A-E). To ensure selection of live Kaede^{+} cells, samples were stained with DAPI (dead cells) and DRAQ5 (live cells) to gate out non-viable cells. A complete negative control (A-A’’’) along with positive controls for Kaede (B-B`), DRAQ5 (C-C’), and DAPI (D-D`) were analyzed to provide a baseline for each sort. Kaede^{-} larvae display little or no fluorescence when excited with the 488nm (A’, Kaede) and 647nm (A’’, DRAQ5) lasers, and some autofluorescence when excited with the UV 450nm laser (A’’’, DAPI). Therefore, dead cells (DAPI^{+}) were excluded based upon their increase in fluorescence from the baseline of 10^4 (E’). DRAQ5^{+}/DAPI^{-} cells (E’) were considered live, and live cells that fluoresce when excited with a 488nm laser were considered Kaede^{+}. (E’’). These cells were immediately sorted into methanol to preserve gene expression, preparatory to sequencing.

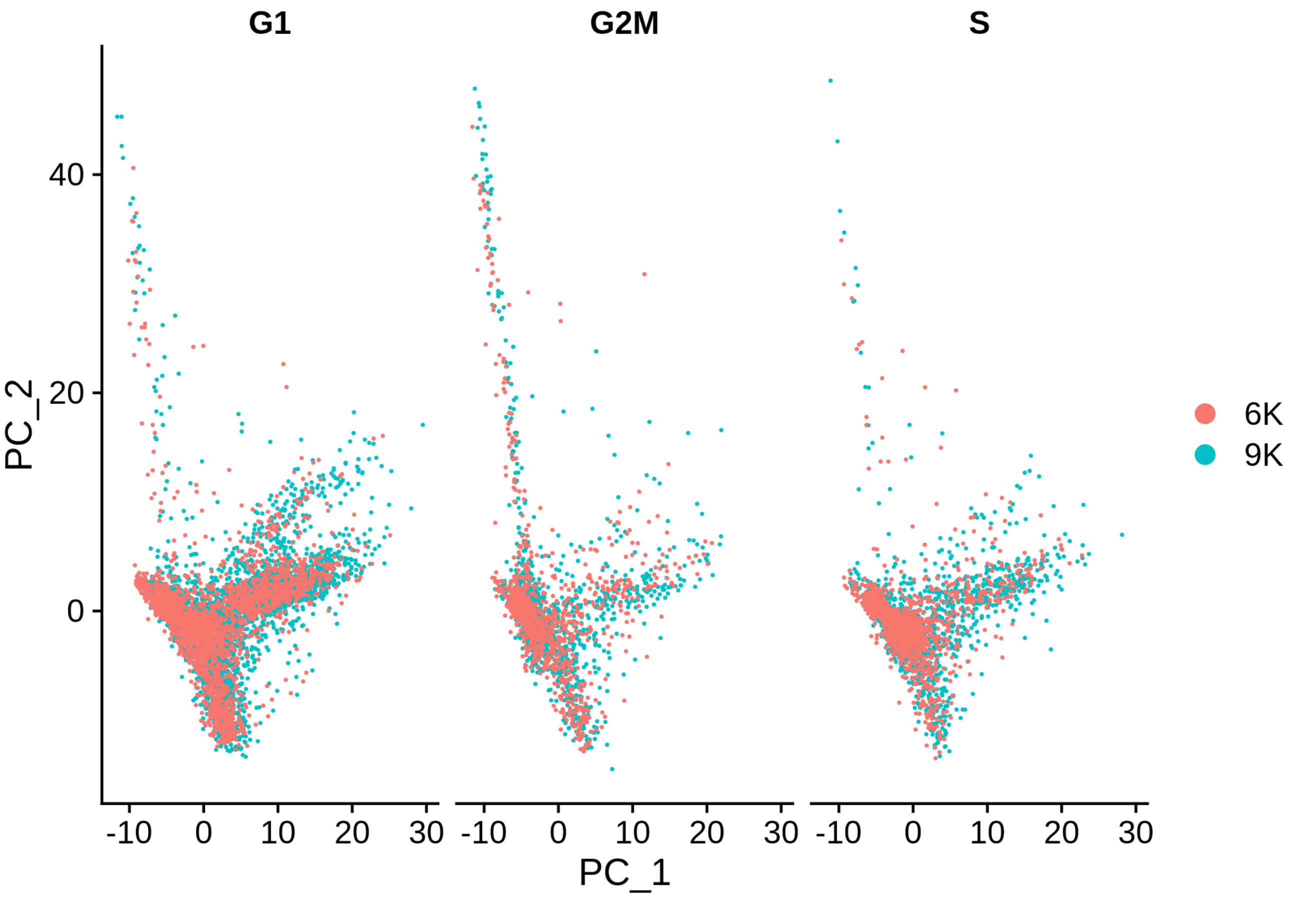

Supplementary Figure 2. Quality Control for Cell Cycle Regression, Related to Methods. Variation in gene expression due to cell cycle can obscure true biological variation. We performed cell cycle scoring for each gene (18222) in the merged dataset to determine if cells group by cell cycle. We found cell cycle did not have an impact on PCA grouping, and regression was not deemed necessary. 6K and 9K denote number of cells sequenced per replicate sequencing replicates

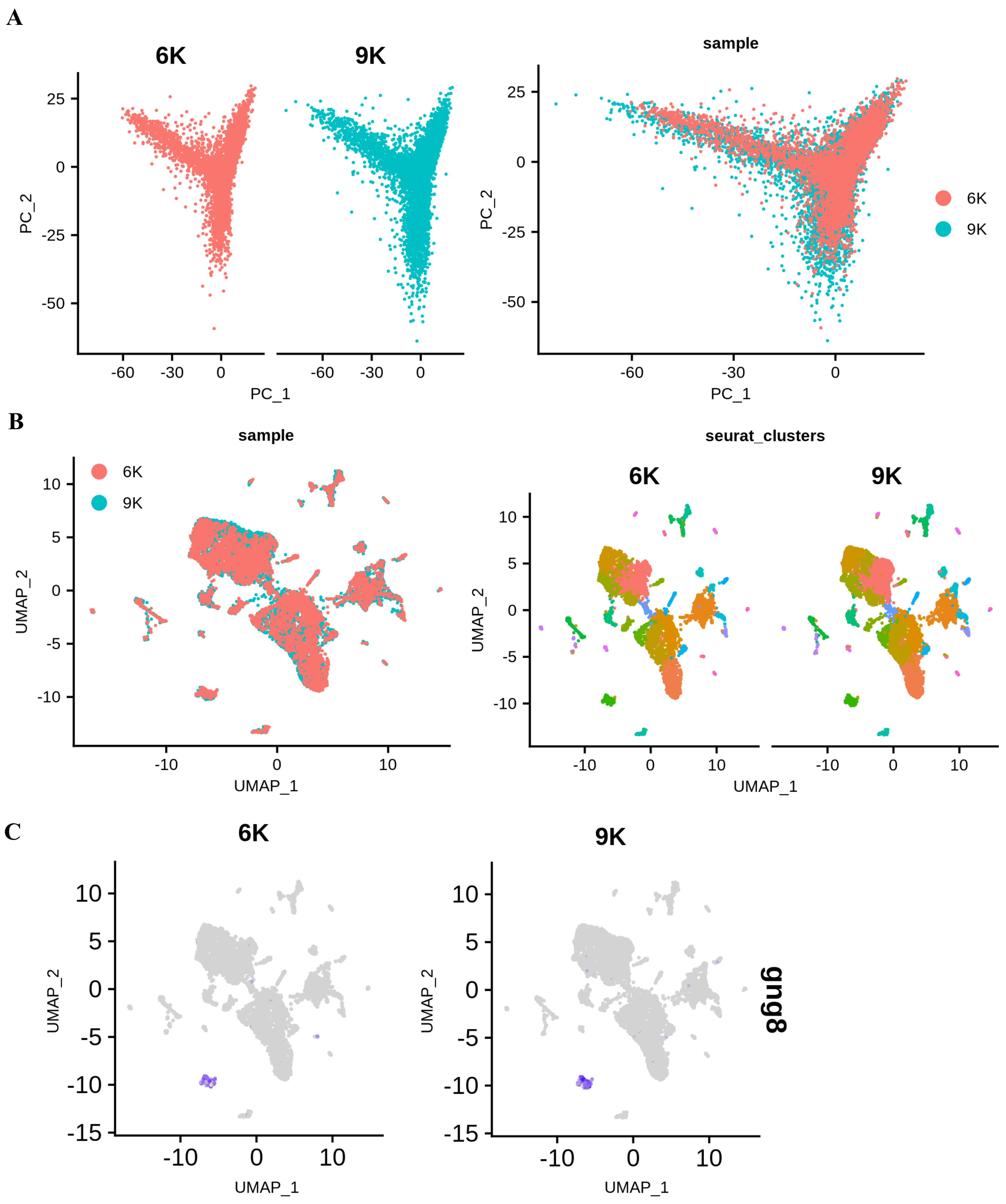

Supplementary Figure 3. Quality Control Check for Batch Effect, Related to Methods. Two temporal replicates of 9,293 and 6,629 cells were used to generate the initial datset of 15,922 total cells; we expect similar variation and do not anticipate batch to obscure meaningful biological variation. (A) PCA plots (left:split by replicate, right: overlay) of each replicate show similar grouping,indicating similar variation. (B) UMAPs of the merged dataset after clustering (left: sample overlay; right: split). (C) UMAPs of the canonical habenula marker gng8 show cells cluster according to cell type rather than replicate, indicating batch effects are not present.

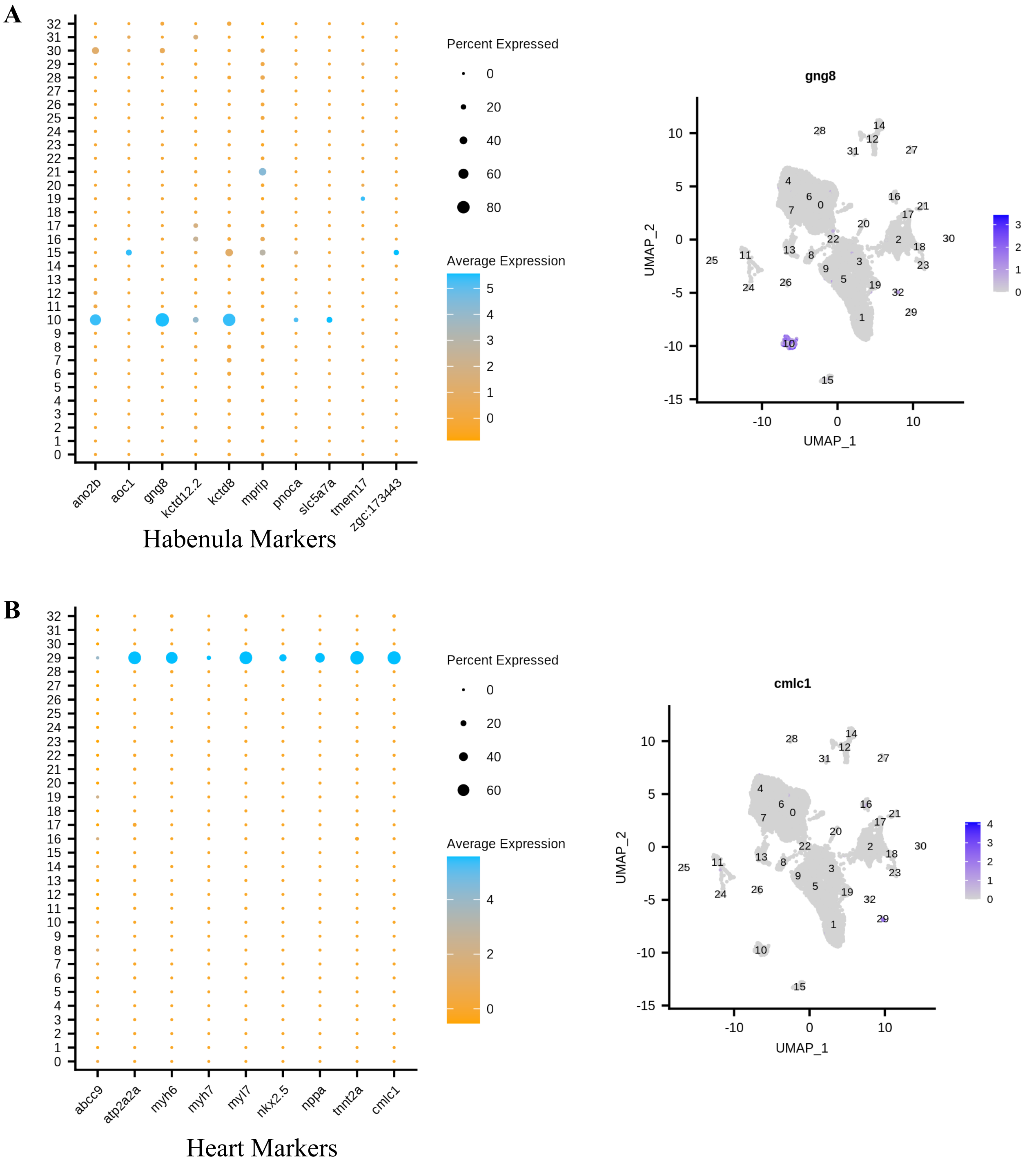

Supplementary Figure 4. Annotation of Habenula and Heart Cells, Related to Figure 1 and Methods. To determine putative tectal cells we used known marker genes to exclude populations of non-interest. (A) (left) Upregulated expression of habenula genes in cluster 10 nominate it as habenular; (right) expression of *gng8*, canonical habenula marker is restricted to cluster 10. (B) Upregulated expression of heart marker genes in cluster 29 nominate it as a heart cluster (left); expression of *cmlc1*, a canonical heart marker, is restricted to cluster 29 (right). See Methods for marker gene curation.

A

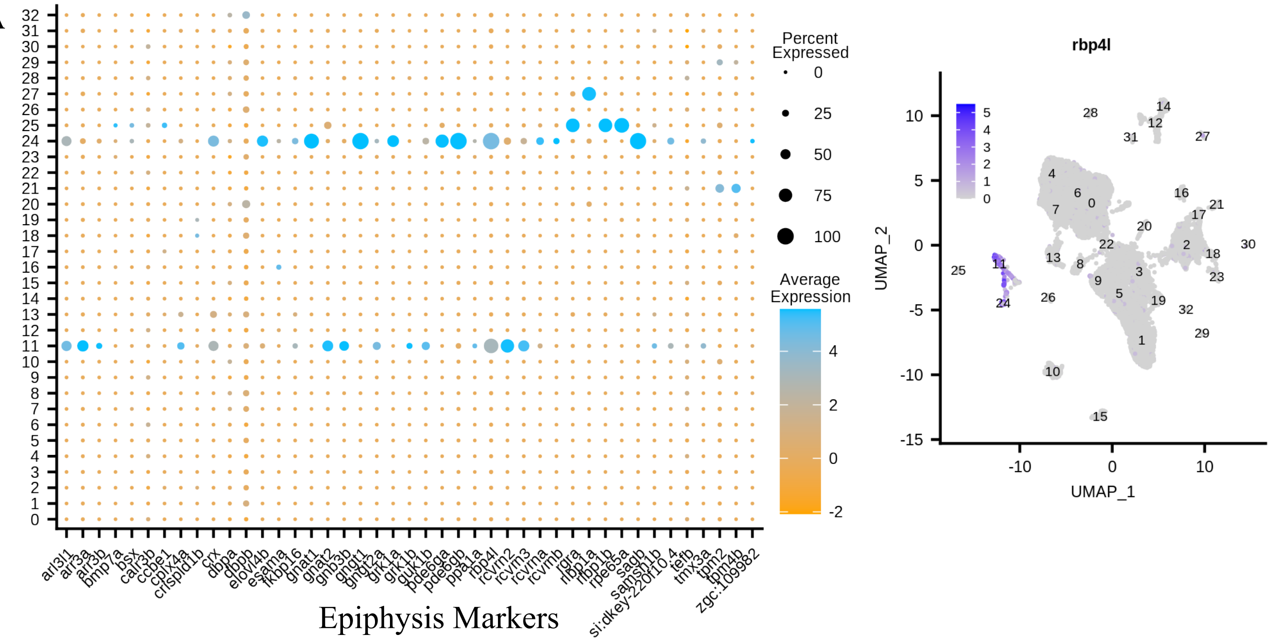

B

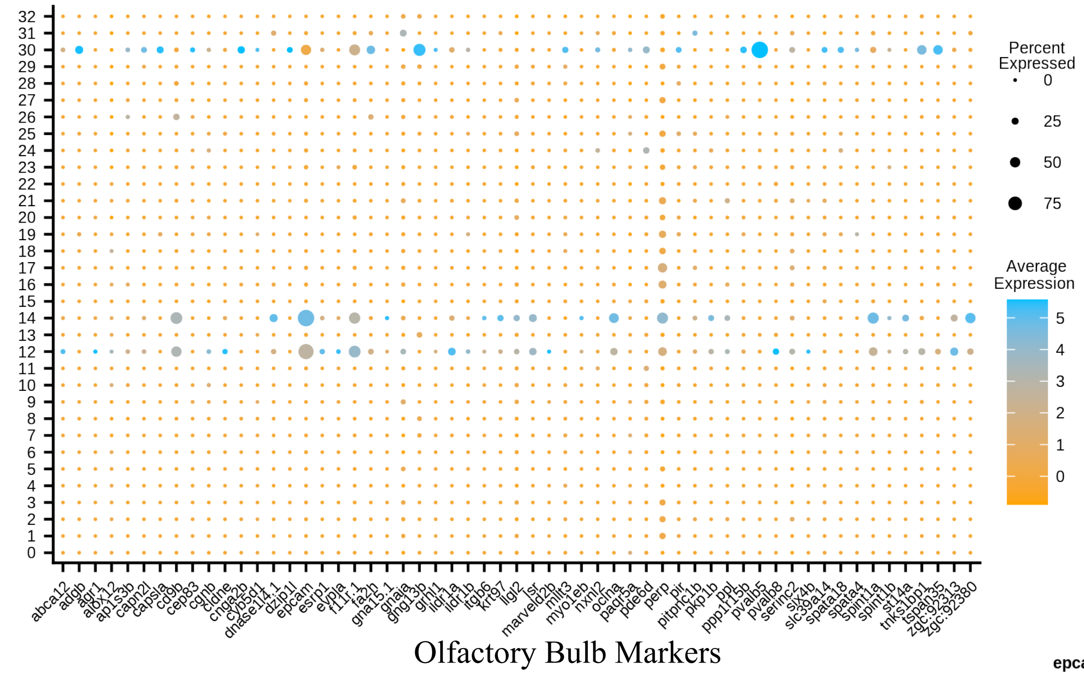

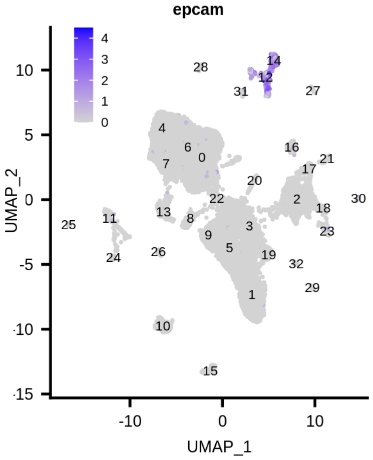

Supplementary Figure 5. Annotation of Epiphysis and Olfactory Bulb Cells, Related to Figure 1 and Methods. To determine putative tectal cells, we used known marker genes to exclude populations of non-interest. (A) (left) Upregulated expression of epiphysis genes in clusters 11/24/25 nominate them as epiphysal; (right) expression of *rbp4l*, canonical epiphysis/ retinal marker is restricted to clusters 11/24/25. (B) Upregulated expression of olfactory genes in clusters 12/14/30 nominate them as olfactory (top left); expression of *epcam*, canonical olfactory bulb marker, is restricted to clusters 12/14/30 (bottom right). See Methods for marker gene curation.

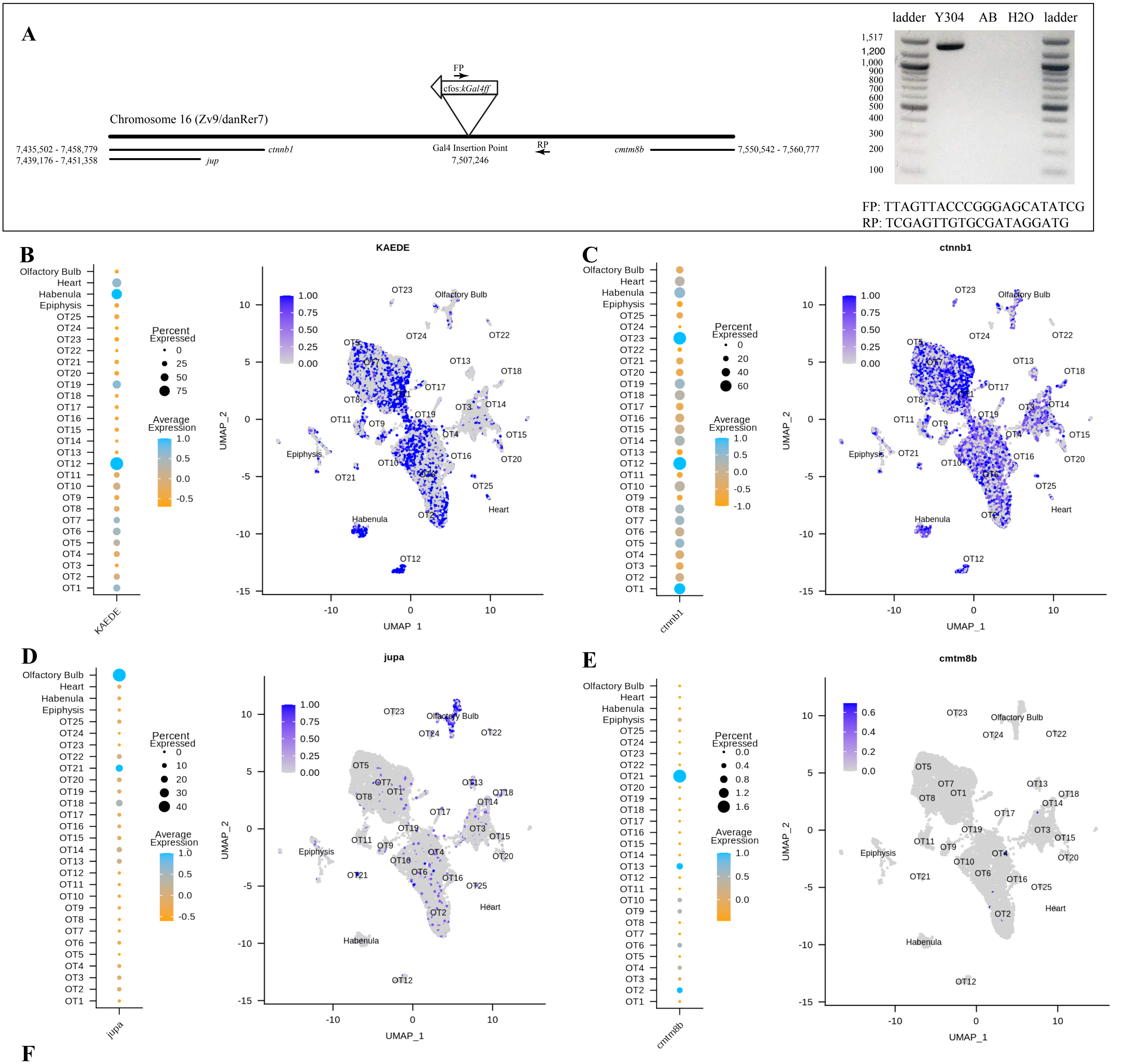

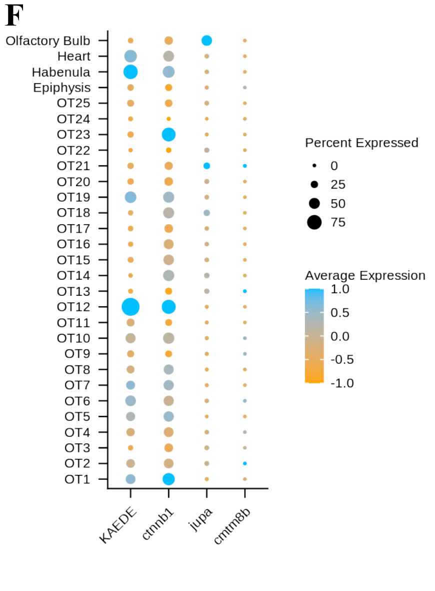

Supplementary Figure 6. Detection of *kaede* and Genes Proximal to *Gal4* Insertion, Related to Methods. PCR amplification confirms that *kGal4ff* is inserted in chromosome 16 between *ctnnb1, jup* and *cmtm8b* in the y304 enhancer trap line (A). No amplification is seen in the wild-type AB line. To confirm FACS isolated cells used for scRNA-seq were labeled by the *y304Et*(cfos:*Gal4*;UAS:*Kaede*) line, we looked for the presence of *kaede* (regulated by the Gal4 bipartite system) and the three closest genes to the *Gal4* insertion site. We reasoned that in addition to *Gal4*, genes proximal to the insertion site are likely modulated by the trapped enhancer. (B-E) DotPlot (left) and graphical UMAP (right) representation showing *kaede* (B), *ctnnb1* (C), *jup* (*jupa*) (D), and *cmtm8b* (E) are present in the FACS isolated and sequenced cells. *kaede* was present in our dataset, although its levels were relatively lower in some of the populations (B). This could be due to a high dropout rate or possible lower mRNA stability in this transgene. *ctnnb1*, a gene previously found to be expressed in areas including OT and habenula (Osborn et al, 2014), is present in all cell populations (with the lowest expression in OT 24) (C). *jup* (*jupa*), previously found to be expressed in the olfactory organ (Thisse et al., 2001; Thisse and Thisse, 2004), is also found in our olfactory cluster (D). *cmtm8b*, predicted to be involved in myelination (ZFIN), is found in OT21, which we designate as an oligodendrocyte population (E & Table 1). (F) DotPlot of the relative expression of the four genes in our dataset.

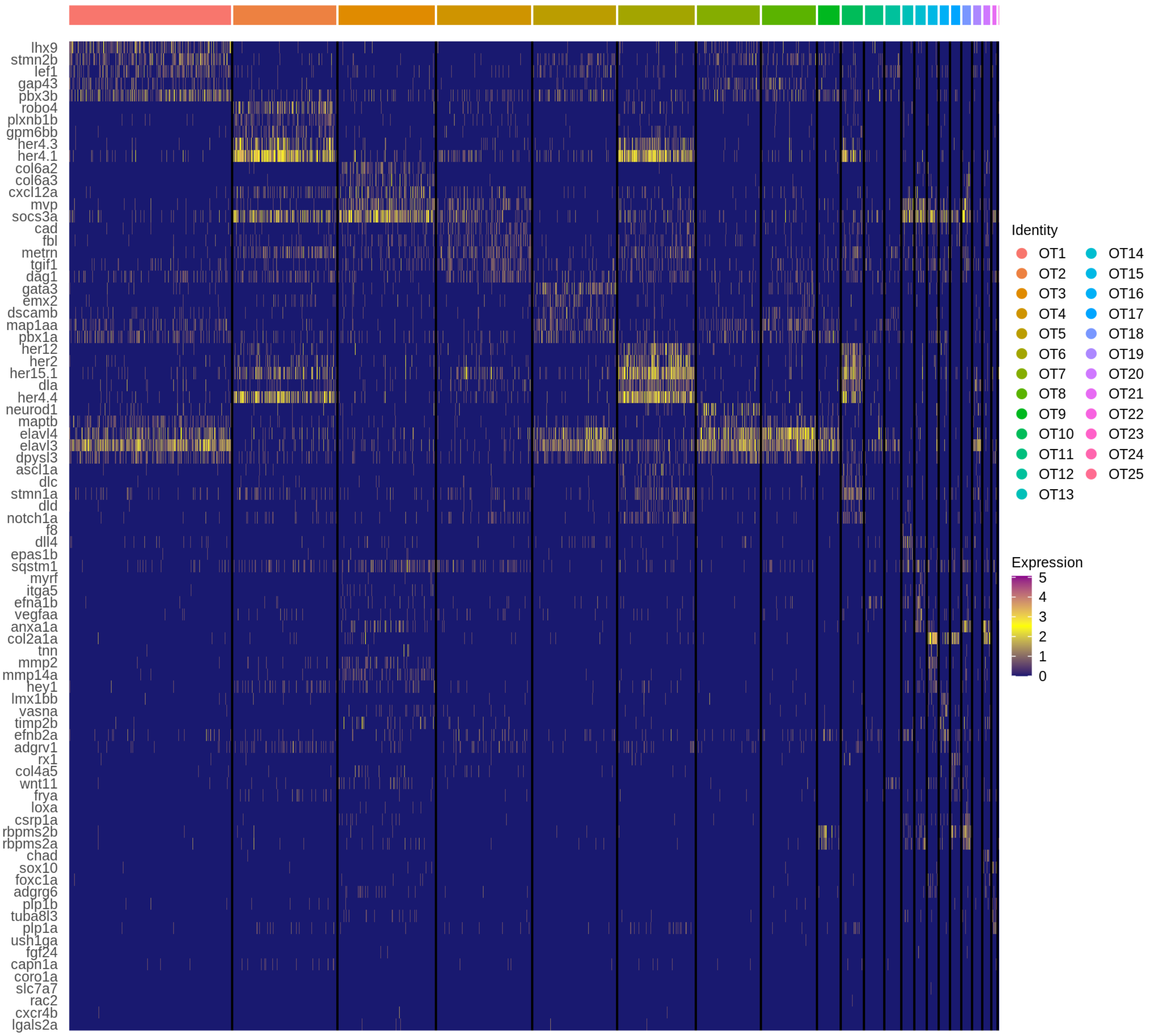

Supplementary Figure 7. Top Five Unique Neurogenesis Genes, Related to Figure 3 and Table 1. Heatmap showing the top five differentially expressed neurogensis genes for each cluster according to log2 fold change; duplicates are allowed but each gene is only represented once. See Methods for differential gene expression parameters.
