# Supplementary material for: Single-Cell RNA Sequencing Characterizes the Molecular Heterogeneity of the Larval Zebrafish Optic Tectum
Source: Front Mol Neurosci. 2022 Feb 10;15:818007. doi: 10.3389/fnmol.2022.818007 (PMC8869500; doi:10.3389/fnmol.2022.818007)
Supplement: Supplementary Table 4 — Mature neuronal markers, related to Figures 3, 4, and methods. [file Table_4.docx]

| Mature Neurons | |
| --- | --- |
| Gene | Function |
| *sv2a* | Predicted to have transmembrane transporter activity. Predicted to be involved in chemical synaptic transmission; neurotransmitter transport; and transmembrane transport. Predicted to localize to cytoplasmic vesicle; integral component of membrane; and synapse. |
| *sv2ba* | Predicted to have transmembrane transporter activity. Predicted to be involved in chemical synaptic transmission and transmembrane transport. Predicted to localize to integral component of membrane; neuron projection; and synaptic vesicle membrane. |
| *sv2bb* | Predicted to have transmembrane transporter activity. Predicted to be involved in chemical synaptic transmission; neurotransmitter transport; and transmembrane transport. Predicted to localize to integral component of membrane; neuron projection; and synaptic vesicle membrane. |
| *sv2a* | Predicted to have transmembrane transporter activity. Predicted to be involved in chemical synaptic transmission; neurotransmitter transport; and transmembrane transport. Predicted to localize to cytoplasmic vesicle; integral component of membrane; and synapse. |
| *sv2ca* | Predicted to have transmembrane transporter activity. Predicted to be involved in chemical synaptic transmission; neurotransmitter transport; and transmembrane transport. Predicted to localize to integral component of membrane; neuron projection; and synaptic vesicle membrane. |
| *vamp1* | Predicted to have SNAP receptor activity and syntaxin-1 binding activity. Predicted to be involved in SNARE complex assembly. Predicted to localize to SNARE complex and plasma membrane. |
| *vamp2* | Predicted to have SNAP receptor activity and syntaxin-1 binding activity. Predicted to be involved in SNARE complex assembly. Localizes to cleavage furrow and vesicle. |
| *vamp3* | Predicted to have SNAP receptor activity and syntaxin-1 binding activity. Predicted to be involved in SNARE complex assembly. Predicted to localize to SNARE complex and plasma membrane. |
| *vamp4* | Predicted to be involved in Golgi ribbon formation; SNARE complex assembly; and microtubule cytoskeleton organization. Predicted to localize to SNARE complex; synaptic vesicle; and trans-Golgi network. |
| *vamp5* | Predicted to have SNAP receptor activity and syntaxin-1 binding activity. Predicted to be involved in SNARE complex assembly. Predicted to localize to SNARE complex and plasma membrane. |
| *vamp8* | Predicted to have SNAP receptor activity and syntaxin binding activity. Predicted to be involved in SNARE complex assembly and mucus secretion. Predicted to localize to SNARE complex; mucin granule; and plasma membrane. |
