# Supplementary material for: Single-Cell RNA Sequencing Characterizes the Molecular Heterogeneity of the Larval Zebrafish Optic Tectum
Source: Front Mol Neurosci. 2022 Feb 10;15:818007. doi: 10.3389/fnmol.2022.818007 (PMC8869500; doi:10.3389/fnmol.2022.818007)
Supplement: Supplementary Table 5 — Glial markers, related to Figure 3 and methods. [file Table_5.docx]

| Glial Markers | | | |
| --- | --- | --- | --- |
| Gene | Link | Expression location | Glial Type |
| *her4.1* | <http://zfin.org/ZDB-GENE-980526-521#expression> | anterior neural rod, ectoderm, nervous system, neural keep, segmental plate | Radial glia |
| *cx43* | <http://zfin.org/ZDB-TSCRIPT-090929-1120> | anterior neural rod, ectoderm, nervous system, neural keep, segmental plate | Radial glia |
| *id1* | <http://zfin.org/ZDB-GENE-990415-96#summary> | brain, ectoderm, germ ring, mesoderm, pleuroperitoneal region | Radial glia |
| *s100b* | <http://zfin.org/ZDB-GENE-040718-290#summary> | digestive system, heart, integument, nervous and renal system. | Radial glia |
| *fabp7a* | <http://zfin.org/ZDB-GENE-000627-1#summary> | gill, nervous system, eye | Radial glia |
| *blbp* | <http://zfin.org/ZDB-GENE-000627-1#summary> | gill, nervous system, eye | Radial glia |
| *glula* | <http://zfin.org/ZDB-GENE-030131-688#summary> | cardiovascular, digestive, hematopoietic, muscular, and nervous system | Radial glia |
| *si:ch211-251b21.1* | <http://zfin.org/ZDB-GENE-060809-5#summary> | central nervous system, proliferative region, spinal cord | Radial glia |
| *fgfbp3* | <http://zfin.org/ZDB-GENE-050208-135#summary> | brain, endoderm, hindbrain, midbrain, telencephalon | Radial glia |
| *atp1a1b* | <http://zfin.org/ZDB-GENE-001212-5#summary> | central nervous system and neural tube | Radial glia |
| *selenop* | <http://zfin.org/ZDB-GENE-030311-1#summary> | head mesenchyme, pronephric duct, yolk, yolk syncytial layer | Radial glia |
| *mdka* | <http://zfin.org/ZDB-GENE-990621-1#summary> | brain, eye, neural tube, neuroectoderm, paraxial mesoderm | Radial glia |
| *slc1a2b* | <http://zfin.org/ZDB-GENE-030131-7779#summary> | nervous system, spinal cord, neural tube | Radial glia |
| *cd82a* | <http://zfin.org/ZDB-GENE-030131-2818#summary> | central nervous system, otic vesicle, pectoral fin, yolk syncytial layer | Radial glia |
| *cxcl12a* | <http://zfin.org/ZDB-GENE-030318-1#summary> | brain, mesoderm, myoseptum, neural rod, sensory system | Radial glia |
| *dhrs12la* | <http://zfin.org/ZDB-GENE-030131-8104#summary> | brain, central nervous system, glial cell, hindbrain | Oligodendrocytes |
| *flj13639* | <http://zfin.org/ZDB-GENE-030131-8104#summary> | brain, central nervous system, glial cell, hindbrain | Oligodendrocytes |
| *mbpa* | <http://zfin.org/ZDB-GENE-030128-2#summary> | EVL, nervous system, otic vesicle, periderm | Oligodendrocytes |
| *mbpb* | <http://zfin.org/ZDB-GENE-030429-21#summary> | nervous system and polster | Oligodendrocytes |
| *mpz* | <http://zfin.org/ZDB-GENE-010724-4#summary> | Rohon-Beard neurons, basal plate midbrain region, central nervous sytem, cranial nerve, oligodendrocytes | Oligodendrocytes |
| *mag* | <http://zfin.org/ZDB-GENE-041217-24#summary> | nervous system | Oligodendrocytes |
| *olig1* | <http://zfin.org/ZDB-GENE-050107-2#summary> | central nervous system, oligodendrocyte, and trunk | Oligodendrocytes |
| *olig2* | <http://zfin.org/ZDB-GENE-030131-4013#summary> | glioblast, nervous system, neural keel, neural plate, and neural tube | Oligodendrocytes |
| *plp1a* | <http://zfin.org/ZDB-GENE-001202-1#summary> | central nervous system, glial cell, nerual tube | Oligodendrocytes |
| *plp1b* | <http://zfin.org/ZDB-GENE-030710-6#summary> | nervous system | Oligodendrocytes |
| *swap70b* | <http://zfin.org/ZDB-GENE-030131-3587#summary> | immature eye, mesoderm, nervous system, pectoral fin, and vasculature | Oligodendrocytes |
| *sox10* | <http://zfin.org/ZDB-GENE-011207-1#summary> | glioblast, head, iridoblast, nervous system, neural crest | Oligodendrocytes |
| *zwi* | <http://zfin.org/ZDB-GENE-030131-8155#summary> | nervous system | Oligodendrocytes |
| *erbb3a* | <http://zfin.org/ZDB-GENE-030916-3#summary> | epidermis, fin, glial cell, heart | Oligodendrocytes |
| *gfap* | <http://zfin.org/ZDB-GENE-990914-3#summary> | anterior neural keel, nervous system, neural tube, neuronal stem cell, and optic vesicle | Oligodendrocytes |
| *dhrs12la* | <http://zfin.org/ZDB-GENE-030131-8104#summary> | brain, central nervous system, glial cell, hindbrain | Oligodendrocytes |
| *mpeg1.1* | <https://zfin.org/ZDB-GENE-030131-7347#summary> | myeloid reporter expressed in tectal microglia | Microglia |
| *slc7a7* | <https://zfin.org/ZDB-GENE-051127-5#summary> | brain microglial cell | Microglia |
| *xpr1b* | <https://zfin.org/ZDB-GENE-060503-266#summary> | brain microglial cell | Microglia |
| *ctsba* | <https://zfin.org/ZDB-GENE-040426-2650#summary> | lysosomal gene enriched in tectal microglia | Tectal microglia |
